# Systematic review of the Exploration, Preparation, Implementation, Sustainment (EPIS) framework

**DOI:** 10.1186/s13012-018-0842-6

**Published:** 2019-01-05

**Authors:** Joanna C. Moullin, Kelsey S. Dickson, Nicole A. Stadnick, Borsika Rabin, Gregory A. Aarons

**Affiliations:** 1 Faculty of Health Sciences, School of Pharmacy and Biomedical Sciences, Curtin University, Kent Street, Bentley, Perth, 6102 Western Australia; 2 Child and Adolescent Services Research Center, 3665 Kearny Villa Rd., Suite 200N, San Diego, CA 92123 USA; 3 Department of Child and Family Development, San Diego State University, 5500 Campanile Drive, San Diego, CA 92182 USA; 4 Department of Psychiatry, University of California San Diego, 9500 Gilman Drive (0812), La Jolla, San Diego, CA 92093-0812 USA; 5 Department of Family Medicine and Public Health, University of California San Diego, 9500 Gilman Drive (0725), La Jolla, San Diego, CA 92093-0812 USA

**Keywords:** Implementation, Framework, Model, Theory, Outer context, Inner context, Process, Systematic review, Diffusion of innovations

## Abstract

**Background:**

Effective implementation of evidence-based practices (EBPs) remains a significant challenge. Numerous existing models and frameworks identify key factors and processes to facilitate implementation. However, there is a need to better understand how individual models and frameworks are applied in research projects, how they can support the implementation process, and how they might advance implementation science. This systematic review examines and describes the research application of a widely used implementation framework, the Exploration, Preparation, Implementation, Sustainment (EPIS) framework.

**Methods:**

A systematic literature review was performed to identify and evaluate the use of the EPIS framework in implementation efforts. Citation searches in PubMed, Scopus, PsycINFO, ERIC, Web of Science, Social Sciences Index, and Google Scholar databases were undertaken. Data extraction included the objective, language, country, setting, sector, EBP, study design, methodology, level(s) of data collection, unit(s) of analysis, use of EPIS (i.e., purpose), implementation factors and processes, EPIS stages, implementation strategy, implementation outcomes, and overall depth of EPIS use (rated on a 1–5 scale).

**Results:**

In total, 762 full-text articles were screened by four reviewers, resulting in inclusion of 67 articles, representing 49 unique research projects. All included projects were conducted in public sector settings. The majority of projects (73%) investigated the implementation of a specific EBP. The majority of projects (90%) examined inner context factors, 57% examined outer context factors, 37% examined innovation factors, and 31% bridging factors (i.e., factors that cross or link the outer system and inner organizational context). On average, projects measured EPIS factors across two of the EPIS phases (*M* = 2.02), with the most frequent phase being Implementation (73%). On average, the overall depth of EPIS inclusion was moderate (2.8 out of 5).

**Conclusion:**

This systematic review enumerated multiple settings and ways the EPIS framework has been applied in implementation research projects, and summarized promising characteristics and strengths of the framework, illustrated with examples. Recommendations for future use include more precise operationalization of factors, increased depth and breadth of application, development of aligned measures, and broadening of user networks. Additional resources supporting the operationalization of EPIS are available.

**Electronic supplementary material:**

The online version of this article (10.1186/s13012-018-0842-6) contains supplementary material, which is available to authorized users.

## Background

Effective implementation of evidence-based interventions, treatments, or innovations (hereafter referred to as evidence-based practices [EBPs]) to address complex and widespread public health issues remains a significant challenge. Our ability to effectively implement an EBP is as important as treatment effectiveness because failed implementation efforts are often the underlying reason for lack of EBP effectiveness or impact in health and social care systems and organizations [1–3]. There are numerous frameworks, models, and theories that identify key factors, and sometimes processes, to facilitate EBP implementation [4–6]. Such implementation frameworks are commonly used to help select and structure research questions, methods, strategies, measures, and results. While an increasing number of studies use implementation frameworks, the ways in which these frameworks are used or operationalized is not well described and their theoretical and practical utility are often left unexamined [7].

The present study is a systematic review of one highly cited and widely used implementation framework, the Exploration, Preparation, Implementation, Sustainment (EPIS) framework [8]. Until recently, this comprehensive framework has had limited prescriptive guidance for its use. The EPIS framework was developed based on examination of the literature on implementation in public sector social and allied health service systems (e.g., mental health, substance use disorder treatment, social care, child welfare) in the USA, and has applicability in other countries and other settings. This study will determine how EPIS has been applied and how widely the framework has been disseminated, adopted, and implemented in diverse health, allied health, and social care sectors, and further afield.

### The EPIS framework

**Fig. 1:**
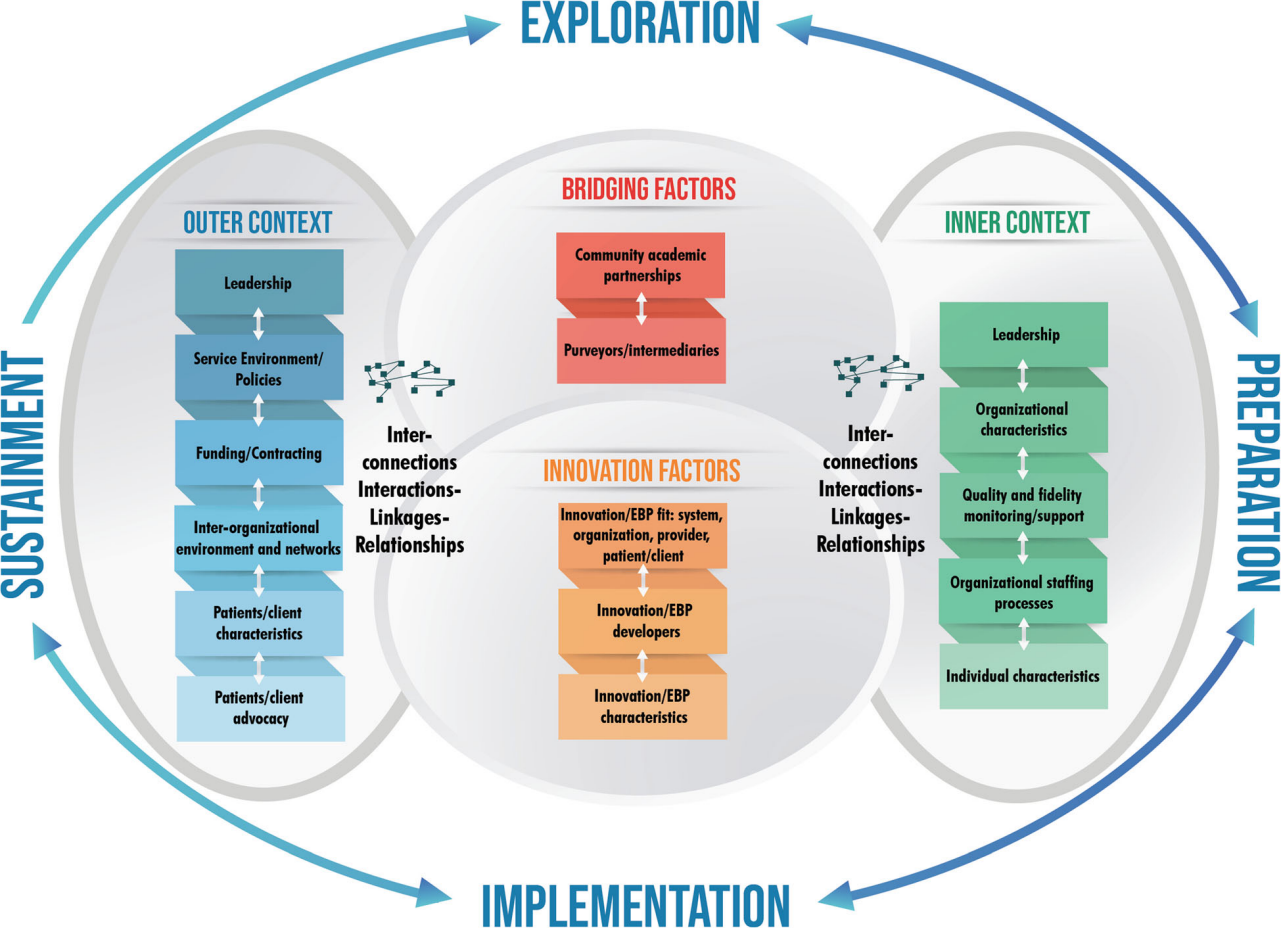
Exploration, Preparation, Implementation, Sustainment (EPIS) framework including phases, Outer/Inner Context, Bridging Factors, and Innovation factors

As shown in Fig. 1, EPIS has key components that include four well-defined phases that describe the implementation process, identification of outer system and inner organizational contexts and their associated factors, innovation factors that relate to the characteristics of the innovation/EBP being implemented, and bridging factors, the dynamics, complexity, and interplay of the outer and inner contexts [8].

The first key component of EPIS is the four phases of the implementation process, defined as Exploration, Preparation, Implementation, and Sustainment (EPIS). In the Exploration phase, a service system, organization, research group, or other stakeholder(s) consider the emergent or existing health needs of the patients, clients, or communities and work to identify the best EBP(s) to address those needs, and subsequently decides whether to adopt the identified EBP. In addition, consideration is given to what might need to be adapted at the system, organization, and/or individual level(s) and to the EBP itself. The exploration phase begins when implementers and relevant stakeholders are aware of a clinical or public health need and are considering ways to address that need. The implementers move into the next phase of preparation upon deciding to adopt one or more EBPs or innovations. In the Preparation phase, the primary objectives are to identify potential barriers and facilitators of implementation, further assess needs for adaptation, and to develop a detailed implementation plan to capitalize on implementation facilitators and address potential barriers. Critical within the Preparation phase is planning of implementation supports (e.g., training, coaching, audit, and feedback) to facilitate use of the EBP in the next two phases (Implementation and Sustainment) and to develop an implementation climate that indicates that EBP use is expected, supported, and rewarded [9]. In the Implementation phase and guided by the planned implementation supports from the Preparation phase, EBP use is initiated and instantiated in the system and/or organization(s). It is essential that ongoing monitoring of the implementation process is incorporated to assess how implementation is proceeding and adjust implementation strategies to support efforts accordingly. In the Sustainment phase, the outer and inner context structures, processes, and supports are ongoing so that the EBP continues to be delivered, with adaptation as necessary, to realize the resulting public health impact of the implemented EBP.

The second key component of the EPIS framework is the articulation of contextual levels and factors comprised of the outer system context and the inner organizational context. Within each phase, outer and inner context factors that could be considered as instrumental to the implementation process are highlighted, many of which apply across multiple implementation phases. The outer context describes the environment external to the organization and can include the service and policy environment and characteristics of the individuals who are the targets of the EBP (e.g., patients, consumers). The outer context also includes inter-organizational relationships between entities, including governments, funders, managed care organizations, professional societies, and advocacy groups, that influence and make the outer context dynamic. For example, collaboration between child welfare and mental health systems may occur, surrounding the development and implementation of a coordinated care program for youth served in both sectors. It is important to note that the outer context is dynamic. The inner context refers to the characteristics within an organization such as leadership, organizational structures and resources, internal policies, staffing, practices, and characteristics of individual adopters (e.g., clinicians or practitioners). Within the inner context, there are multiple levels that vary by organization or discipline and may include executive management, middle management, team leaders, or direct service providers (e.g., clinicians, practitioners). Together, the inner and outer contexts reflect the complex, multilayered, and highly interactive nature of the socioecological context of health and allied healthcare that is noted in many implementation frameworks [8, 10–12].

The third key component of EPIS are the factors that relate to the EBP or innovation itself. There is an emphasis of fit of the EBP to be implemented with the system and patient/client population (outer context), as well as the organization and provider (inner context). This implies that some adaptation to the EBP will likely be necessary. The aim is to maintain the core components on an EBP and adapt the periphery.

The fourth and final component of EPIS is the recognition of the interconnectedness and relationships between outer and inner context entities, what is part of what we refer to as bridging factors. The bridging factors are deemed to influence the implementation process as the inner context of organizations is influenced by the outer system in which the organization operates, but those influences are reciprocal (e.g., industry lobbyists impacting pharmacy legislation, and direct to consumer marketing). For example, hospitals are subject to federal, state, and local policies that define certification and reporting requirements.

EPIS considers that adaptation (often involving implementation strategies) will likely be necessary in regard to the outer and inner contexts as well as to the EBP. This is supported by recent work identifying the need for a dynamic approach to adaptation that involves all relevant stakeholders through the four EPIS phases in order to capitalize on the knowledge and experience of the implementation team and maximize the ability to find solutions that are acceptable to all stakeholders [13]. Furthermore, this is consistent with calls for consideration of the need for adaptation in EBP sustainment [14, 15]. This emphasis on adaptation to improve fit within the EPIS framework is akin to what others have identified as fostering values-innovation fit [16, 17]. The values-innovation fit proposes that innovation implementation will be more successful if there is a high degree of fit between the values and needs of implementers and the characteristics of the innovation to be implemented [16]. For example, one implementation strategy may be to develop system and organizational climates that support such a values-innovation fit [18]. EPIS also explicitly identifies the importance of EBP characteristics and the role of EBP developers and purveyors/intermediaries (i.e., those who support the implementation process) throughout the process of implementation and demonstration of effectiveness. This is especially important when considering values-innovation fit and identifying potential adaptations to increase EBP fit within a specific setting while preserving fidelity to EBP core elements that are responsible for clinical or service outcomes.

It is unclear the degree to which these varying components of EPIS are identified, operationalized, and studied in the published literature. To address this gap, we conducted a systematic review of the literature to describe how the EPIS implementation framework has been used in peer-reviewed, published studies. This review (1) describes EPIS use in implementation research to date and (2) makes recommendations for using the EPIS framework to advance implementation science theory, research, and practice.

## Methods

A multi-step process was used to identify, review, and analyze the use of the EPIS framework.

### Search strategy

A systematic search of the literature was executed in May 2017 to locate studies published in academic journals that utilized the EPIS framework. The search strategy was based on cited reference searching of the original EPIS article titled “Advancing a Conceptual Model of Evidence-Based Practice Implementation in Public Service Sectors” [8]. The title was used as the TITLE search term in each database. The following seven databases and search criteria were used: (1) PubMed single citation matcher (TITLE in ALL FIELDS CITED by), (2) Scopus: (TITLE in REFERENCES), (3) PsycINFO: (TITLE in REFERENCE and PEER REVIEWED), (4) ERIC: (TITLE in ANYWHERE), (5) Web of Science: (TITLE AND THE “TIMES CITED” LINK FOR FULL LIST OF CITATIONS OF THE ORIGINAL EPIS PAPER), (6) Social Sciences Index (TITLE in All Text Fields), and (7) Google Scholar: (TITLE Cited By).

Our initial search criteria were anchored to the original EPIS citation [8] to ensure complete identification of articles that had used the EPIS framework. We utilized the title of the original article—rather than the more recently accepted acronym that follows from the EPIS phases. As such, all records were published between 2011 (when the EPIS framework was published) and 2017 (when the search was conducted).

Prior to assessment of inclusion and exclusion criteria duplicates of resulting articles were removed.

### Inclusion and exclusion criteria

Inclusion criteria were as follows:

1. Report on an original (empirical) research study
2. Published in a peer-reviewed journal
3. Study design or implementation project focused on dissemination, implementation, or sustainment, including hybrid designs.
4. Utilized the EPIS framework to guide study theory, design, data collection, measurement, coding, analysis, and/or reporting

Papers were excluded if they were conceptual (e.g., commentary, debate) rather than empirical research or a synthesis (e.g., EPIS was cited as one of a list of frameworks, theories, or models but was not used in a meaningful way).

### Data collection

Four reviewers (JM, KD, NS, BR) assessed titles, abstracts, and full articles for inclusion. Each article was independently assessed by two reviewers. Papers (*n* = 74; 9.7%), where there was a difference of opinion regarding inclusion, were assessed by a third reviewer (GA).

### Data extraction

Each article was critically appraised by two reviewers independently. Reviewers extracted the data summarized in Table 1 from each included article. Refer to Additional file 1 for extracted data of each article.

**Table 1:** Data extraction

Author	List of authors
Year	Year of publication
Objective	Summary of publication’s objective(s)
Country	Country where implementation efforts were conducted
Setting	Physical setting where implementation took place (e.g., mental health clinic, church, community center, primary care)
Sector	Sector (e.g., psychology, social work, mental Health, behavioral health, public health)
EBP, Innovation or Intervention	Specific EBP (i.e., the innovation or intervention) implemented
Health focus	Whether a health focus was reported (yes/no)
Study design	Study design as reported in the paper (e.g., prospective, retrospective, hybrid implementation, case study, participant observation)
Study methodology	Study methodology (i.e., qualitative, quantitative, or mixed)
Larger Study Design	Methodology of larger study, if the effort was part of a larger study
Type of EPIS use	How EPIS was used (e.g., study design, data collection, measurement, analysis, coding, and/or reporting/interpretation)
Level of data collection	Level(s) of data collection (e.g. outer context, inner context, multilevel)
Level of analysis	Level(s) of analysis (e.g., provider, team, supervisor, organization, system)
Outer context	Whether outer context factors were assessed (yes/no)
Inner context	Whether inner context factors were assessed (yes/no)
Innovation factors	Whether innovation factors were assessed (yes/no)
Bridging factors	Whether other bridging factors within EPIS were assessed (yes/no)
Implementation strategy	Whether there was a researcher (co)initiated implementation strategy (yes/no) and reported (yes/no)
Implementation outcomes	Implementation outcomes (e.g., feasibility, adoption, fidelity)
Stages	EPIS phase(s) in which implementation factors were assessed: Exploration, Preparation, Implementation, Sustainment; Phase(s) were rated for the degree to which the authors were explicit in their use, where 0 = phase not included, 1 = implicit inclusion of phase, 2 = explicit inclusion of phase. Explicit inclusion is where the authors overtly included the named phase(s) of EPIS that were included in their study, while implicit inclusion was assessed by the reviewers based on the EPIS phase definitions
Depth	Overall depth of inclusion of EPIS, from 1 = conceptual (e.g., inner and outer context factors were applied to study design but not carried through the study and evaluation phases) to 5 = operationalized (e.g., looked at a few factors incorporated throughout the paper [intro, design, measurement, conclusions] or included all phases)

Additional classifications of implementation factors from the original 2011 paper were added to the data extraction and EPIS framework figure. There was limited emphasis in the original 2011 paper and also a large presence of factors regarding the innovation and factors connecting the innovation and the two contexts, which highlighted the need for additional terms to better represent particular factors that fall outside of the inner and outer context. As such, we refer to these as Innovation factors (for example innovation/EBP characteristics, innovation/EBP fit), and Bridging factors, or factors that span the inner and outer contexts (for example, interagency collaboration and community-academic partnerships).

### Synthesis of results

Data were recorded in an extraction table (Additional file 1). Following data extraction, each reviewer met with the paired team member of each article to compare their results, reach consensus for areas that required further discussion or review, and combine extracted data into a single entry in the data extraction table. Finally, studies that had multiple articles were grouped together based on the parent project. In Additional file 1, projects that contained more than one article have a summary of the project reported first, followed by articles within each parent project listed in italics.

## Results

**Fig. 2:**
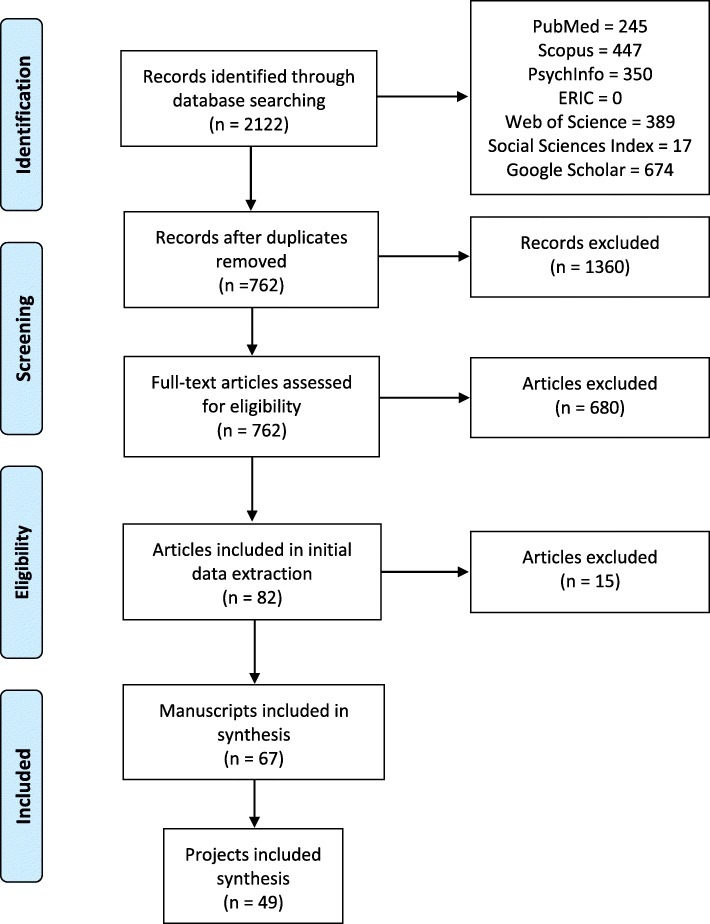
PRISMA Flow Diagram of paper selection [62]

Our search identified 2122 records (Fig. 2). After removal of duplicates, 762 articles were screened (title, abstract, and full-text review as needed) of which 67 initially met the inclusion criteria. Articles were then grouped by projects (if projects had one or multiple articles) resulting in a total of 49 unique research projects (see Additional file 1). Further results are reported by project.

Projects utilizing EPIS were conducted in 11 countries that span high-, low-, and middle-income contexts: USA (39 projects, plus 2 in collaboration with other countries), Canada (1 project in collaboration with USA), Mexico (1 project), Sweden (1 project), Norway (1 project in collaboration with USA), Belgium (1 project), Australia (2 projects), UK (1 project), Brazil (1 project), the Bahamas (1 project), and South Africa (1 project). In 47% of projects, one or more authors had a professional connection (e.g., sponsor or mentor on a training grant) or research collaboration (e.g., co-investigator on a grant, co-author on a scholarly product) with the lead author of the original EPIS framework paper, Dr. Gregory Aarons; 6% and 4% had a professional connection or collaboration with the second (Hurlburt) and third (Horwitz) authors, respectively.

The included projects spanned a variety of public sectors: public health, child welfare, mental or behavioral health, substance use, rehabilitation, juvenile justice, education, and school-based nursing. The child welfare and mental health sectors had the highest representation across the projects (13 and 19 projects, respectively). The physical setting for the projects varied from whole service systems (e.g., Mental Health Department) to organizational level settings (e.g., schools, child welfare agencies, substance use treatment programs, community-based organizations, health clinics). The scale of the projects ranged from case studies of a few organizations to very large studies across hundreds of organizations or several states and counties. All projects were in public sector settings.

The majority of projects (*n* = 36, 73%) were investigating the implementation of one specific EBP. Two projects offered sites a selection of EBPs (4%), while 11 projects (22%) were implementing the concept of EBP. The health focus was reported in 8 projects (16%). These included (but not limited to) maltreatment, behavioral problems, mental health or substance use, human immunodeficiency virus (HIV), Parkinson’s Disease, teen pregnancy, and workplace disability.

There was a reasonable division in the methodologies employed in the projects. Eleven projects (22%) used quantitative methods, 13 (27%) qualitative methods, and 26 (53%) mixed methods. Projects often produced separate articles focusing on qualitative and quantitative methodology, with 22 (32%) quantitative articles, 21 (31%) qualitative articles, and 25 (37%) mixed-methods articles. The data collected in the projects included assessment of multiple inner context levels (20 projects, 41%), followed by assessment of one inner context level (15 projects, 31%) and then assessment of multiple outer and inner levels (14 projects, 29%). Data analyses conducted in the projects was usually multilevel. In total, only 12 projects (24%) were analyzed at a single level. Seven study protocols were included in the review, two used EPIS only in the study design, while three had subsequent results articles included. Seven projects employed EPIS only in analysis or coding, and/or reporting. Four projects used EPIS to frame the study and then in reporting, but not in study design, data collection or measurement. Six projects (12%) used EPIS only as background to frame the study. The role of EPIS in the projects has been summarized in Table 2.

**Table 2:** Use of EPIS in included projects and articles

	Projects	Articles
Study design	37	28
Reporting	37	28
Framing	34	23
Measurement	24	18
Data collection	23	18
Analysis	18	14
Coding	13	10

*Note*: Categories are not mutually exclusive. Included projects and articles may have used the EPIS for multiple purposes

In terms of the outer and inner context, innovation, and bridging factors, we found some variation in reporting. Factors associated with the outer and inner context were reported in 57% (*n* = 28) and 90% (*n* = 44) of projects, respectively. A large number of projects included innovation factors (37%) and bridging factors that spanned the inner and outer contexts (31%).

Regarding the EPIS phases, we noted a variation in how explicitly projects (i.e., authors overtly included the name of the phases) identified the various phases and differences in what phases were included in projects. Table 3 summarizes the distribution of the EPIS phase(s) examined and whether the phase was explicitly versus implicitly used. All of the included projects and the majority of the articles (78%) explicitly focused on the Implementation stage whereas a minority of the projects (29%) and articles (23%) explicitly focused on the Exploration stage. On average, projects included 2.02 out of the four phases (SD = 1.14). Table 4 reports the frequency of EPIS factors at each phase measured in the included articles. The most frequently measured factors across phases were organizational characteristics and individual adopter characteristics.

**Table 3:** EPIS stage used in included projects and articles

	Projects explicit	Projects implicit	Articles explicit	Articles implicit
Exploration	14	4	14	5
Preparation	17	5	18	9
Implementation	38	4	49	9
Sustainment	18	5	23	6

*Note*: Phases are not mutually exclusive. A specific project or article may have focused on multiple phases

**Table 4:** Frequency of EPIS factors in each phase

	Constructs	Phases			
		E	P	I	S
Outer context	Service environment	6	9	17	10
	Policies	2	6	12	6
	Funding/contracting	8	12	20	14
	Leadership	2	2	7	6
	Inter-organizational environment and networks	9	8	15	12
	Client characteristics	2	2	4	3
	Patient/client advocacy	1	0	2	1
Innovation factors*	Innovation/EBP developers	0	0	0	0
	Innovation/EBP characteristics	1	1	1	1
	Innovation/EBP fit*	5	6	14	9
Bridging factors*	Community-academic partnerships	1	1	2	2
	Purveyors/intermediaries	0	0	0	0
Inner context	Organizational characteristics	13	18	32	22
	Culture	4	6	11	5
	Climate	7	9	13	6
	Leadership	6	9	22	9
	Quality and fidelity monitoring/support	2	4	8	3
	Organizational staffing processes	7	9	27	15
	Individual characteristics	16	24	34	19
	Attitudes towards EBPs	9	10	17	9
	Demographics	4	5	7	4

*Represents factors that are new and not defined in original EPIS 2011 paper

Frequency counts represent unique articles

Finally, to quantify the overall coverage of the EPIS framework, a rating from 1 (conceptual use of EPIS) to 5 (operationalized use of EPIS) was assigned. The average rating of EPIS depth was 2.8 out of 5, indicating a moderate depth of EPIS application.

## Discussion

This review describes how one of the most commonly used D&I frameworks [7], the EPIS framework, has been used in practice and reported in the literature since its first publication in 2011 until mid-2017. A total of 49 unique research projects using EPIS, published in 67 peer-reviewed articles, were identified. Projects were conducted in 11 countries, across a range of public sector settings. While the EPIS framework was developed based on the literature on implementation in public social and allied health service systems in the USA, it appears to have broad applicability in other countries and other health and/or allied health settings.

The promise of implementation science models and frameworks is that they may allow for cross-setting and cross-country comparison of factors associated with implementation, which can contribute to our understanding of optimal implementation strategies and generalizability of concepts and constructs, support the harmonization of measures and evaluation practices, and help advance the field of implementation science and implementation practice. This review shows the promise and utility of EPIS to guide studies in various settings, topic areas, and geopolitical locations, and economically resourced regions. For example, our results demonstrate that EPIS has been used in high-, low-, and middle-income countries including Sweden [19], South Africa [20], and Mexico [21]. EPIS has also been used in other settings including public health [22], schools [23], and community health centers [24]. We encourage adaptation and use of EPIS outside of currently tested projects.

There may be some tendency for frameworks, models, and theories to be used by those in aligned information and professional networks [25]; however, it is likely that a given framework may have broad applicability across settings. The EPIS framework is a relatively young model compared to some other implementation models and frameworks in the field. It is natural that after initial introduction and application, the network of users will broaden [26]. This has already been observed with EPIS, as more than 50% of research projects included in this review had no direct affiliation with the first author of the EPIS framework (Aarons). We expect that this natural diffusion of EPIS will continue and will be enhanced as more diverse examples of its use emerge. Moreover, we anticipate that more comprehensive use of EPIS, including such aspects as inter-organizational networks, innovation fit at system, organization, provider, and patient levels, may be enhanced through the examples, recommendations, and resources described in this review [27–30].

EPIS was developed as a comprehensive, stand-alone implementation framework containing the core components [4] of implementation; the implementation process was divided into four phases, and an enumeration of potential influencing factors for each phase across the multilevel context may be evaluated quantitatively and qualitatively, allowing for the testing of related implementation strategies. We reviewed the completeness and frequency with which the key components of EPIS have been used across research projects. The depth of EPIS inclusion was moderate. However, we recommend more in-depth use in the articulation, application, and measurement of factors included in implementation frameworks. On the other hand, use of all components of a framework is not always feasible, practical, desirable, or necessary for a given implementation study or project [31] and many implementation frameworks do not include all the core components of implementation [4].

In terms of the process-related characteristics of the EPIS phases (i.e., moving through phases of Exploration, Preparation, Implementation, and Sustainment), we found variability in the use of process-related aspects of EPIS, with the most frequent phase being Implementation. Furthermore, the majority of the research projects had the Implementation phase as their main focus with much less emphasis on the Exploration, Preparation, and Sustainment phases. This finding is consistent with other literature suggesting that thoughtful planning which could happen in the Exploration and Preparation phases for implementation and sustainment is infrequent although critical [4]. It is also documented in the literature that attention to sustainment is sparse but is imperative for ongoing and meaningful change, improvement in outcomes, and public health impact [32, 33]. We suggest that implementation researchers begin with sustainment in mind and as the ultimate goal of implementation efforts. This perspective does not preclude, and even embraces, the need for careful navigation through Exploration, Preparation, and Implementation phases and for adaptations of the outer or inner contexts, implementation strategies, and the EBP(s). Examples of the use of EPIS in the Exploration and Preparation phase include projects that examine service providers and supervisors/administrators attitudes towards and knowledge of an EBP(s) to inform implementation strategies and adaptation efforts [34, 35].

Projects in this review varied in regard to the depth with which EPIS was described and operationalized, with only some cases of EPIS being applied throughout the implementation phases. For the most benefit, it is desirable that implementation models and frameworks are used to inform all phases of the research process from early development of implementation research questions through to presentation and dissemination of research findings. It is also true that frameworks might have diverse strengths and might be more appropriate to use for certain purposes than others. There are five broad categories that frameworks have been classified into based on their primary purpose: process models, determinant frameworks, classic theories, implementation frameworks, and evaluation frameworks [5, 36]. For example, the Reach, Effectiveness, Adoption, Implementation, and Maintenance Framework (RE-AIM) has historically been used as a planning and evaluation framework [37, 38] and the Consolidated Framework for Implementation Research (CFIR) is frequently used as a determinant to guide qualitative methods and analyses [39]. EPIS can be classified in many categories as it may be used for the purpose of understanding process, determinants, implementation, and evaluation. By guiding multiple components of implementation, the EPIS framework may be used for several purposes, reducing the need for use of multiple frameworks.

It is critical to go beyond the mention of the framework in the introduction of a research grant or paper or only applying the framework retrospectively during data analysis, without sufficient operationalization of the framework in the research process. A content review of U.S. National Institutes of Health grant proposal abstracts, funded and not funded, showed that one key characteristic of funded proposals was that the implementation framework(s) selected was better described and operationalized [40]. We recommend careful consideration and operationalization of components, and also relating use of theory in testing and advancing knowledge of what aspects of implementation frameworks are more or less useful in driving implementation process and advancing implementation science. Greater depth and breadth of EPIS use would include providing descriptions of the implementation plan, the factors included in the project, and how and when the specified factors and process are being assessed.

There was variability in the specific factors examined at each phase, although organizational and individual adopter characteristics were the most frequent factors across all phases. It is not surprising to see that inner context factors are most commonly assessed. The relative higher frequency of measuring organizational and individual adopter characteristics may be influenced by the greater availability of quantitative measures of these factors in comparison to system level factors (refer to Table 5 for list of associated measures). A recent publication in the journal *Implementation Science* highlighted the need to better define and develop well operationalized and pragmatic measures for the assessment of external implementation context and bridging factors [41]. Access to existing measures is provided through a number of resources and publications [42–44]. More specifically, measures for various EPIS factors have been developed and tested through a number of studies. Examples of these measures are provided in Table 5. Development and use of additional measures meeting these criteria is a high priority area for Implementation Science.

**Table 5:** Examples of quantitative measures of EPIS factors used in published studies

	Sample of EPIS factors	Example quantitative measures
Outer context	Service environment	Sustainability assessment tool
	Policies	EBP-specific document review (i.e., speeches, regulations, annual reports; documented system-wide policy)
	Funding/contracting	–
	Leadership	Leadership Competence Scale of Program Sustainability Index [63]
	Inter-organizational environment and networks	–
	Patient/client characteristics*	Demographics, Administrative Claims Data
	Patient/client advocacy*	–
Innovation factors*	Innovation/EBP developers	–
	Innovation/EBP characteristics	–
	Innovation/EBP fit*	–
Bridging factors*	Community-academic partnerships*	–
	Purveyors/intermediaries	–
Inner context	Organizational characteristics	Group Innovation Inventory [64]; Implementation Climate Scale [65]; Level of Institutional Scale [66]; Organizational Climate Measure [67]; Organizational Culture and Climate via Children’s Services Survey [68]; Organizational Readiness for Change [69]; Organizational Social Context Survey [70]; Organizational Size; Program Sustainability Index [71]; Siegel Scale of Support of Innovation [72]
	Culture	Organizational Culture and Climate via Children’s Services Survey [68]; Organizational Social Context Survey [70]
	Climate	Implementation Climate Assessment [66]; Implementation Climate Scale [65, 72]; Organizational Climate Measure [67]; Organizational Social Context Survey [64]; Time Climate Inventory [73]
	Readiness for change	Organizational Readiness for Change [69]; Readiness for Organizational Change [74]
	Leadership	Implementation Leadership Scale [75]; Multifactor Leadership Questionnaire [76]
	Quality and fidelity monitoring/support*	Adherence and Skill Checklist [77]; Assessment of Climate Embedding Mechanisms [18]; Examination of Common Dimensions of EBI(s); performance-based role-plays [78]; Therapist Procedures Checklist-Revised [79]; Therapist Procedures Observational Coding System [80]
	Supportive coaching	Coaching records
	Organizational staffing processes	Data regarding turnover rates and reasons
	Individual characteristics	Demographics; Emotional Competency Inventory [81]; Evidenced-Based Practice Attitudes Scale [82]; Knowledge of Evidenced-Based Services Questionnaire [83]; organizational readiness for change [69]
	Attitudes towards EBPs	Evidence-Based Practice Attitudes Scale [82]; Perceived Characteristics of Intervention Scale; Barriers to Research Practice Scale [84]
	Implementation citizenship*	Implementation Citizenship Behavior Scale [61]
	Burnout*	Maslach Burnout Inventory [85]

*Represents factors that are new and not defined in original EPIS 2011 paper

Examples are only provided for those factors that were measured in the review—indicates that there were no quantitative measures in the included articles of this review

It is important to note that the role and relevance of factors within the inner and outer context might vary across phases. Some factors might be important throughout all phases (e.g., funding, organizational characteristics), while others might have heightened relevance during one or two of the phases (e.g., quality and fidelity monitoring/support during the Implementation and Sustainment phases). We also emphasize the importance of attending to the bridging factors and the dynamic interplay between inner and outer context factors. We encourage those using the EPIS framework to use theory or a logic model of their particular implementation strategy and context to decide what factors are likely to be critical and relevant in their study [45, 46]. Detailed and deep use of implementation models and frameworks to identify specific implementation determinants and targets, and processes of implementation can help to address these concerns. The model developed from the EPIS framework for the Interagency Collaborative Team (ICT) project provides an example of interconnectedness and relationships between and within outer and inner context entities [47]. In the ICT project, a community-academic partnership was formed to bridge the outer and inner contexts. Furthermore, interagency collaborative relationships within and across the contextual levels were formed including between outer context policy makers with advocacy groups and community-based organizations contracted to provide home-based services with clients and families [48]. Outer context policies were instantiated through collaborative processes such as community stakeholder meetings, the use of negotiations, and procurement and contracting. Contracts, which clearly specifies the expectation to use EBPs, communicates a strong system level support (outer context) for a climate (inner context) where EBPs are expected, supported, and rewarded [49].

As discussed, EPIS includes levels across the socioecological context [12], touching on factors at the individual, organizational, and systems levels. A multi-level conceptualization of implementation processes, and the understanding that interactions across various levels need to be considered, has been an increasing discourse in the Implementation Science literature [10]. A strength of EPIS is in its perspective that draws attention to the complexities of its multi-level conceptualization including data collection and data analysis. For example, when collecting qualitative data the interviewer may ask about the respondent experience at their own unit level (e.g., experience of supervisors in their team) or other levels (e.g., the larger agency or system level policies). It is important to specify hypotheses both within levels and across levels. As an example, interventions to improve leadership and organizational implementation climate may be intended to improve clinician attitudes towards EBP, and EBP adoption, use, and fidelity [18]. In this case, the higher level leadership and climate are at the higher unit level, while attitudes, adoption, and fidelity are at the individual clinician level.

The multi-level contextual nature of EPIS lends itself to a variety and integration of methodologies using quantitative only, qualitative only, or mixed-method approaches. There is an increasing appreciation in Implementation Science for the need to use a combination of quantitative and qualitative methods which allow for a more complete assessment of our often context-dependent, pragmatic research questions (i.e., for whom, under what circumstances, why, and how does an intervention or implementation strategy work) [50, 51]. In our review, we found a number of examples where mixed-methods approaches guided by the EPIS framework were able to provide more comprehensive evaluation of an implementation research problem. For example, Gleacher and colleagues [52] used qualitative interview data from clinicians to augment quantitative utilization and implementation data to examine multilevel factors associated with adoption and implementation of measurement feedback systems in community mental health clinics. A critical challenge in the field is to find ways to publish findings from mixed-method studies; we found that two thirds of the mixed-method projects in this review published their qualitative and qualitative findings in separate papers. Space limitations and orientation of various journals (i.e., more qualitative or quantitative focus) might form barriers for mixed-methods findings to be published in an integrated manner. There are resources on how to apply mixed-methods to Implementation Science research that give guidance and examples of integration of qualitative and quantitative conceptualization, data collection and analysis, and reporting [53–55].

## Future directions of EPIS

The results from this systematic review have informed our recommendations for the future use of EPIS for (1) more precise operationalization of EPIS factors, (2) consideration of the interplay between inner and outer context through bridging factors, and (3) discussion of how EPIS can be consistently incorporated with greater depth and throughout the lifespan of an implementation project (breadth).

### Recommendation no. 1: Precise operationalization of EPIS factors

The use of precise and operationalized definitions of EPIS factors is key to facilitate the successful application of this framework and guide appropriate measurement of factors. In this vein, we have refined definitions of the EPIS factors (see Table 6). The definitions are flexible to ensure applicability of EPIS factors across phases and multiple levels. For example, the inner context factor *organizational characteristics* is defined as “structures or processes that take place or exist in organizations that may influence the process of implementation.” Inherent within this definition is that this construct may be an important consideration within any of the four EPIS phases and at multiple levels (e.g., provider, team, supervisor). Moving forward, we encourage implementation scientists to utilize these definitions to inform their application and measurement of EPIS factors, as well as using the EPIS factors and relationships between factors to develop theoretical models for testing in implementation studies.

**Table 6:** Definitions of EPIS factors

	EPIS constructs	Definition	Examples
Outer context	Service environment/policies*	State and federal sociopolitical and economic contexts that influence the process of implementation and delivery/use of the innovation	Policies; legislation; monitoring and review; auditing; mandates
	Funding/contracting	Fiscal support provided by the system in which implementation occurs. Fiscal support can target multiple levels (e.g., staff training, fidelity monitoring, provision of the innovation/EBP) involved in implementation and delivery/use of the innovation	Contracting arrangements; grants; fee-for service, addition to formulary; capitation fees, incentives
	Leadership	Characteristics and behaviors of key decision-makers pertinent at all levels who are necessary but not sufficient to facilitate or promote the implementation process and delivery/use of the innovation	Transformational leadership; Implementation leadership
	Inter-organizational environment and networks	Relationships of professional organizations through which knowledge of the innovation/EBP is shared and/or goals related to the innovation/EBP implementation are developed/established	Inter-organizational collaboration, commitment, competition, co-opetition
	Patient/client characteristics*	Demographics and individual characteristics of the target population/end user	Socioeconomic status, health condition, comorbidities, age, gender, motivation
	Patient/client advocacy*	Support or marketing for system change based on consumer needs, priorities and/or demographics	Client advocacy; class-action lawsuits, consumer organizations
Innovation factors*	Innovation/EBP developers	Characteristics of the individuals or team(s) responsible for the creation of the EBP/innovation that may be the subject of implementation efforts	Engagement in implementation, continuous quality improvement, rapid-cycle testing, prototyping
	Innovation/EBP Characteristics	Features or qualities of innovations to be implemented	Complexity, ease of learning, cost, burden, reporting requirements
	Innovation/EBP fit*	The extent to which the innovation/EBP fits the needs of the population served or context in which it is implemented	Innovation/EBP structural and process fit with system, organizations, providers, patients/clients
Bridging factors*	Community-academic partnerships*	Active partnerships between researchers and key community stakeholders, who can represent multiple levels involved in implementation (e.g., system representatives, organizational leaders, providers, consumers), that can facilitate successful implementation and delivery/use of the innovation	Community participation; partnerships; ongoing positive relationships; valuing multiple perspectives
	Purveyors/intermediaries	Organizations or individuals providing support or consultation for implementation and/or training in the innovation	Implementation readiness assessment, strategy development, training support
Inner Context	Organizational characteristics	Structures or processes that take place and/or exist in organizations that may influence the process of implementation	Culture; climate; readiness for change; structure; leadership; receptive context; absorptive capacity; social network support
	Leadership	Characteristics and behaviors of individuals involved in oversight and/or decision-making related to EBP implementation within an organization	Competing priorities; use of climate/culture embedding mechanisms; transformational leadership; implementation leadership
	Quality and fidelity monitoring/support*	Processes or procedures undertaken to ensure adherence to active delivery of the innovation/EBP and/or an implementation strategy	Fidelity support system; quality assurance evaluation; continuous quality improvement
	Organizational staffing processes	The processes or procedures in place at an organization related to the hiring, review, and retention of staff involved in the active delivery of the innovation/EBP and/or its implementation	Professional training and qualification related to EBI delivery; staff turnover
	Individual characteristics	Shared or unique characteristics of individuals (e.g., provider, supervisor, director) that influence the process of implementation	Attitudes towards EBP; demographics and/or background; client characteristics; job demands

*Represents factors that are new or adaptations based on the original EPIS 2011 paper

### Recommendation no. 2: Consideration of the dynamic interplay between inner and outer context factors

In addition to inner and outer context factors, we also now explicitly highlight and define the integral role of bridging factors. These factors were previously conceptualized as those that interlace the inner and outer context factors but were not formally classified within the EPIS framework (see Fig. 1 of Aarons et al. [2011] paper) [8]. In our current conceptualization, these factors and their interactions include: *Community Academic Partnerships, and Purveyors/Intermediaries*. For example, the Dynamic Adaptation Process [13] incorporates an explicit emphasis on these bridging factors to inform EBP adaptation in a planned, systematic way to increase its feasibility for implementation and sustainment. As our results suggest, these bridging factors are active ingredients to aid in understanding the interaction between outer and inner context factors and thus represent a key area of consideration in future work.

### Recommendation no. 3: Increase EPIS depth and breadth

Our results show that more than one phase and level of EPIS have been considered in many implementation studies, highlighting the breadth of the EPIS framework. While this is encouraging, we recommend that future implementation efforts consider how EPIS can be applied longitudinally throughout *all* phases (i.e., Exploration, Preparation, Implementation and Sustainment) and levels (e.g., system, organization, provider) of the implementation process. We suggest that implementation efforts “begin with sustainment in mind.” This reflects the increasing emphasis within implementation science on explicit incorporation or acknowledgement of the sustainment phase from the outset of study planning and execution [56, 57]. Further, our results suggest that EPIS was most commonly used to inform the study design, report results, and frame the research endeavor. We recommend that EPIS, as a theoretical framework, be thoughtfully applied throughout a project from study framing to explicit identification of how EPIS was used within various levels of data collection and analysis and through reporting and interpretation of results. In a longitudinal study design, factors may be evaluated across multiple EPIS phases. Examples of quantitative measures are provided in Table 5 and definitions for qualitative analyses in Table 6.

Finally, the phases of the implementation process may be operationalized by defining and measuring movement through the phases. For example, when an organization is aware of or shows interest in using an EBP, they enter the Exploration phase. Subsequently if they make the decision to adopt the EBP then they would move into the Preparation phase. First use of the EBP would signify transition into the Implementation phase. Lastly, continued use over a designated period of time may be defined as being in Sustainment. These types of movements have been flagged for incorporation into guidelines such as PRECIS-2 [58].

### Exemplar of comprehensive use of EPIS framework: JJ-TRIALS

One example of meticulous and comprehensive use of EPIS is the US National Institute on Drug Abuse (*NIDA)* Juvenile Justice Translational Research on Interventions for Adolescents in the Legal System (*JJ*-*TRIALS*) project [59, 60]. In this major multiple center (six centers, 34 study sites) cluster randomized implementation trial, EPIS was used throughout the implementation phases and across contextual levels to stage the implementation process, select quantitative and qualitative measures, and identify important outcomes. JJ-TRIALS is probably the best and most explicit example of application of the EPIS framework. Indeed, JJ-TRIALS may be one of the best examples of a rigorous, deep, and thoughtful approach to applying an implementation science framework to a large-scale implementation initiative. For example, JJ-TRIALS is testing a bundled strategy that supports the implementation of data-driven decision-making using two different facilitation approaches (core and enhanced). JJ-TRIALS moves beyond implementation of a single EBP to allow for implementation of evidence-based process improvement efforts. Activities to move through the EPIS phases were mapped out along with implementation factors and appropriate measures and strategies to assess and address the key multilevel system and organizational issues. Ways to document and evaluate the implementation factors, implementation strategies, and movement through all of the EPIS phases were determined. In addition, there was a conceptual adaptation of EPIS itself based on input and perspectives of community partners, investigators, and NIH staff wherein the framework was represented in a more recursive and cyclical manner consistent with improvement processes and this resulted in the development of EPIS Wheel [59, 60]. As shown in Fig. 1, based on our current systematic review, we have also provided a depiction of the EPIS framework using a more cyclical perspective that also captures the key features of outer context, inner context, as well as the nature of the practice(s) to be implemented (innovation factors), and the interaction with intervention developers and purveyors that may foster appropriate adaptations of context and practice (bridging factors).

### EPIS resources

#### EPIS website: episframework.com

The website https://EPISFramework.com provides a number of tools for planning and use of EPIS throughout the implementation process. The website is now available and is a living resource that will be continually updated and enhanced.

## Limitations

There are several limitations of this systematic review. We limited the review to peer-reviewed, empirical articles citing Aarons et al. 2011. Ongoing or completed grant-funded studies or contracts that applied EPIS are not included. In addition, unpublished applications of EPIS would not have been included nor articles that do not directly cite Aarons et al. 2011, or articles without searchable reference citations. As such, our results likely do not reflect all implementation efforts that used EPIS and in particular the search strategy may have limited the inclusion of practitioners’ application of the framework for implementation practice. Our rating of the depth to which EPIS was used was based on one item that was developed by the study team. Although operationalized and internally consistent as used in this study, it was not a standardized measure of EPIS use.

## Conclusion

The EPIS framework has a great promise to serve as a multilevel, context-sensitive, broadly applicable framework for Implementation Science research and practice. Our review described the patterns of use to date, summarized promising characteristics and strengths of the EPIS framework, and illustrated those through examples. We also provide recommendations for future use including more precise operationalization, increased depth and breadth of EPIS application, improved use measures for a number of factors, and the ongoing broadening of networks of users, topics, and settings. Additional resources supporting the operationalization of EPIS are available and under development [61].

## Additional file

Additional file 1: Data Extraction. (PDF 1250 kb)
