# Supplementary material for: Systematic review of the Exploration, Preparation, Implementation, Sustainment (EPIS) framework
Source: Implement Sci. 2019 Jan 5;14:1. doi: 10.1186/s13012-018-0842-6 (PMC6321673; doi:10.1186/s13012-018-0842-6)
Supplement: Supplementary file 1 — Data Extraction. (PDF 1250 kb) [file 13012_2018_842_MOESM1_ESM.pdf]

Additional File 1: Data Extraction

Project	Authors	Year	Objective	Country	Setting	Sector	EBP, Innovation or Intervention	Health focus reported (y/n)	Study design	Study methodology	Larger Study Design	Type of EPIS use	Level of data collection	Level of analysis	Outer context (y/n)	Inner context (y/n)	Innovation factors (y/n)	Bridging factors (y/n)	Imp Strategy (y/n)	Imp Strategy Reported (y/n)	Implement. Outcomes	Exploration	Preparation	Implementation	Sustainment	Depth (1: conceptual to 5: operational)
1			Examine the statewide implementation of an EBI. Study looked at impact on key provider characteristics, the influence of leadership and leader member exchange on implementation climate and use of mixed-methods as a methodology for implementation research.	USA	Statewide network of private nonprofit community based mental health and social service provider organizations in one US state	Child welfare	SafeCare[a]	N	Prospective, longitudinal 2 x 2 hybrid design RCT	Mixed methods		Study framing, measurement, analysis, reporting	Multiple level outer and inner context	System, organizational, supervisors, provider	N	Y	N	N	Y	Y	Fidelity, implementation, maintenance	0	0	2	1	**3**
	Aarons GA, Fettes DL, Sommerfeld DH et al.[1]	*2012*	*Mixed Methods for Implementation Research: Application to Evidence-Based Practice Implementation and Staff Turnover in Community-Based Organizations Providing Child Welfare Services*	*USA*	*Statewide network of private nonprofit community based mental health and social service provider organizations in one US state*	*Child welfare*	*SafeCare[a]*	*N*	*Prospective, implementation RCT*	*Mixed method*		*Study framing, study design*	*Multiple level outer and inner context*	*System, organizational, supervisors, provider*	*N*	*Y*	*N*	*N*	*Y*	*Y*	*Fidelity*	*0*	*0*	*2*	*1*	**3**
	Aarons GA, Sommerfeld DH.[2]	*2012*	*Examine the influence of leadership and leader member exchange on implementation climate and staff attitudes towards EBPs during a statewide implementation of SafeCare.*	*USA*	*30 teams providing home-based services to families in a statewide child-welfare system in one US state*	*Child welfare*	*SafeCare[a]*	*N*	*Cross-sectional survey*	*Quantitative*		*Study design, measurement, analysis, reporting*	*Single level inner context*	*Organizational*	*N*	*Y*	*N*	*N*	*N*	*N*	*N*	*0*	*0*	*2*	*0*	**2**
	Green AE, Cafri G, Aarons G.[3]	*2016*	*Examine the impact of a statewide implementation of an EBI (SafeCare) on key provider characteristics, including job autonomy and work attitudes.*	*USA*	*Statewide network of private nonprofit community based mental health and social service provider organizations in one US state*	*Child welfare*	*SafeCare[a]*	*N*	*2 x 2 hybrid implementation trial*	*Quantitative*		*Study framing, measurement, interpretation of findings*	*Single level inner context*	*Organizational, provider*	*N*	*Y*	*N*	*N*	*Y*	*Y*	*N*	*0*	*0*	*2*	*0*	**3**
2	Aarons GA, Green AE, Palinkas LA *et al.*[4]	2012	Protocol to compare the effectiveness of two implementation strategies (traditional versus dynamic adaptation process) for the implementation of Safe Care, a behavioral and psychosocial EBP developed to prevent child neglect.	USA	Child-welfare and home-visitation county services in multiple counties in one US state	Child welfare	SafeCare[a]	N	Prospective Sequential Design (Hybrid Type 3 Implementation Design)	Mixed method		Study framing, study design, data collection, measurement, analysis, reporting	Multiple level outer and inner context	System, organization, provider, client	Y	Y	N	N	Y	Y	Fidelity, feasibility, acceptability, utility	2	2	2	2	**5**
3			To examine factors impacting the sustainment of an EBI across several public service sectors following multi-state, state-wide hybrid-design implementation trial	USA	Home-based services in 11 systems in 87 counties across 2 US states	Child welfare	SafeCare[a]	N	Prospective and retrospective, observational mixed methods study using surveys, interviews	Mixed Method		Study framing; study design, data collection; measurement; data analysis; reporting	Multilevel across outer and inner contexts	System, organization and provider	Y	Y	Y	Y	Y	N	Fidelity, adoption, reach/ penetration, maintenance	0	0	2	2	**5**
	Aarons GA, Green AE, Willging CE et al.[5]	*2014*	*Mixed-method study of a conceptual model of evidence-based intervention sustainment across multiple public-sector service settings*	*USA*	*11 service systems in 87 counties*	*Multiple public service sectors (mental health, public health, social services)*	*SafeCare[a]*	*N*	*Retrospective and Prospective*	*Mixed method*		*Study framing; study design, data collection; measurement; data analysis; reporting*	*Multilevel across outer and inner contexts*	*System, organization and provider*	*Y*	*Y*	*N*	*Y*	*Y*	*N*	*Fidelity, adoption, reach/ penetration, maintenance*	*0*	*0*	*1*	*2*	**5**
	Willging CE, Green AE, Gunderson L et al.[6]	*2015*	*Study how policymakers at the system level pave the path for EBP implementation and influence ongoing utilization within public sector services.*	*USA*	*Social Work and Child Welfare Agencies*	*Child welfare*	*SafeCare[a]*	*N*	*Semi-structured interviews*	*Qualitative*		*Study design, data collection, coding, reporting*	*Single level outer context*	*System*	*Y*	*N*	*Y*	*Y*	*N/A*	*N*	*N*	*0*	*0*	*2*	*2*	**4**

Project	Authors	Year	Objective	Country	Setting	Sector	EBP, Innovation or Intervention	Health focus reported (y/n)	Study design	Study methodology	Larger Study Design	Type of EPIS use	Level of data collection	Level of analysis	Outer context (y/n)	Inner context (y/n)	Innovation factors (y/n)	Bridging factors (y/n)	Imp Strategy (y/n)	Imp Strategy Reported (y/n)	Implement. Outcomes	Exploration	Preparation	Implementati	Sustainment	Depth (1: conceptual to 5: operational)
	Aarons GA, Green AE, Trott E et al.[7]	2016	Examine how outer and inner context leadership were related to system-wide EBI sustainment.	USA ----	Home-based services in 11 systems in 87 counties across 2 US states	Child welfare	SafeCare[a]	N	Prospective, longitudinal, observational, mixed methods using surveys and interviews	Mixed method		Study framing; data collection; measurement; data analysis; reporting	Multilevel across outer and inner contexts	System and team	Y	Y	N	N	Y	N	Fidelity	0	0	1	2	**4**
	Willging CE, Aarons GA, Trott EM et al.[8]	2016	Examine implementation of the Best Value-Performance Information Procurement System to contract for EBIs in a child welfare system, including the impact of key contextual factors.	USA	Home visits	Child welfare	SafeCare[a]	N	Iterative Case Studies using semi-structured interviews	Qualitative		Study design, data collection, coding, and reporting	Multilevel outer context	System	Y	Y	Y	Y	N/A	N	N	0	0	2	2	**4**
4			Examine the role of collaboration in a large-scale countywide implementation of an EBP.	USA	Community-based organizations delivering home-based services	Child welfare	SafeCare[a]	N	Longitudinal study	Mixed method		Study framing, study design, coding, reporting	Multilevel outer and inner context	System, organization, provider	Y	Y	Y	Y	Y	Y	Fidelity; acceptability; sustainability	2	2	2	2	**4**
	Aarons GA, Fettes DL, Hurlburt MS et al.[9]	2014	Examine the role of collaboration in a large-scale countywide implementation of an EBP.	USA	Community-based organizations delivering home services in one County in one US state	Child welfare	SafeCare[a]	N	Descriptive, semi-structured interviews and/or focus groups	Qualitative		Study framing, study design, coding, reporting	Multilevel outer and inner context	System, organization, provider	Y	Y	Y	Y	Y	Y	N	1	1	2	0	**4**
	Chaffin M, Hecht D, Aarons G et al.[10]	2016	Test whether an interdisciplinary collaborative team strategy transmits fidelity to subsequent provider cohorts and examine the impact of a culturally adapted version on cultural competency and fidelity.	USA	Community-based organizations delivering home-based services	Child welfare	SafeCare[a]	N	prospective implementation study using growth modelling to compare fidelity trajectories	Quantitative		Study framing	Single level inner context	Provider	Y	Y	Y	Y	Y	Y	Fidelity	0	2	2	1	**3**
	Hurlburt M, Aarons GA, Fettes D et al.[11]	2014	Describe an implementation strategy (ICT model) and present preliminary results of its challenges across a service system implementing SafeCare.	USA	Community-based organizations delivering home-based services	Child welfare	SafeCare[a]	N	Observational – in-depth interviews	Qualitative		Study framing, study design, reporting	Multilevel across outer and inner contexts	System, organization, supervisor, team, provider, client	Y	Y	Y	Y	Y	Y	N	2	2	2	2	**4**
	Willging CE, Trott EM, Fettes D et al.[12]	2017	Examine the impact of outer and inner context factors, namely funding, fidelity requirements, provider demand and time, impacted the implementation and sustainment of SafeCare.	USA	Home visits/Child Welfare (multiple Child Welfare agencies across one large county in one US state)	Child welfare	SafeCare[a]	N	prospective, qualitative design	Qualitative		Study framing; coding; reporting	Multilevel inner context	Organization, provider	Y	Y	Y	Y	Y	Y	Fidelity; acceptability; sustainability	0	0	2	2	**4**
5	Aarons GA, Ehrhart MG, Moullin JC et al.[13]	2017	Protocol to test the effects of leadership and organizational change for implementation (LOCI) in facilitating motivational interviewing (MI) implementation in substance use disorder treatment (SUDT) settings.	USA	Substance use disorder treatment programs across one US state	Substance use	Motivational interviewing (MI)[b]	N	multiple cohort, cluster randomized trial	Quantitative		Study design, measurement, analysis	Multilevel inner context	Workgroup/ Team	N	Y	N	N	Y	Y	Y	0	2	2	0	**4**
6	Ault-Brutus A, Lee C, Singer S et al.[14]	2014	Examine factors important for delivering a patient activation/self-management intervention.	USA	13 mental health clinics across six US states and territories	Mental health	DECIDE[c]	N	process evaluation - interviews	Qualitative	Hybrid trial	Study design, data collection, coding, reporting	Single level inner context	Provider	N	Y	N	N	Y	N	N	0	0	2	0	**3**

Project	Authors	Year	Objective	Country	Setting	Sector	EBP, Innovation or Intervention	Health focus reported (y/n)	Study design	Study methodology	Larger Study Design	Type of EPIS use	Level of data collection	Level of analysis	Outer context (y/n)	Inner context (y/n)	Innovation factors (y/n)	Bridging factors (y/n)	Imp Strategy (y/n)	Imp Strategy Reported (y/n)	Implement. Outcomes	Exploration	Preparation	Implementati	Sustainment	Depth (1: conceptual to 5: operational)
7			Study the impact of a policy from the Department of Behavioral Health encouraging and incentivizing providers to implement evidence-based treatments to treat youth with mental health problems, with moderating impact of key contextual factors.	USA ----	Large urban public mental health system in one large US city	Mental health	EBPs for mental health (e.g. cognitive behavioral therapy)	Y	prospective, sequential design with multiple observations, surveys and a hybrid-design case-study	Mixed method		Study framing, study design, data collection, measurement, analysis, coding, reporting	Multilevel inner context	Organizational, supervisor, provider	Y	Y	Y	Y	Y	Y	Adoption, reach/ penetration, fidelity	1	2	2	1	**4**
	Beidas RS, Aarons G, Barg F et al.[15]	*2013*	*Policy to implementation: Evidence-based practice in community mental health - study protocol*	*USA*	*30 community mental health clinics in one large US city*	*Mental health*	*EBPs for youth and adults with mental health difficulties*	*N*	*prospective, sequential design with multiple observations*	*Mixed method*		*Study framing, study design, data collection, measurement, analysis, coding, reporting*	*Multilevel inner context*	*Organizational, supervisor, provider*	*Y*	*Y*	*N*	*Y*	*Y*	*N*	*Fidelity, adoption*	*0*	*2*	*2*	*0*	***3***
	Beidas RS, Stewart RE, Adams DR et al.[16]	*2016*	*Examine stakeholder perspectives of implementation facilitators and barriers across contextual levels impacting the implementation and sustainment of EBPs following publicly mandated EBP initiative in a large, urban public mental health system.*	*USA*	*Large urban public mental health system in one large US city*	*Mental health*	*Evidence-based mental health interventions*	*N*	*cross-sectional*	*Qualitative*		*Study framing, data collection, coding, interpretation of findings*	*Multilevel outer and single inner context*	*All*	*Y*	*Y*	*Y*	*Y*	*Y*	*Y*	*N*	*0*	*0*	*2*	*1*	***4***
	Beidas RS, Adams DR, Kratz HE et al.[17]	*2016*	*Case study of efforts to implement a trauma-informed system in large publicly-funded behavioral health system, including key implementation strategies and outcomes.*	*USA*	*Publicly Funded Behavioral Health System for Children and Adolescents in one large US city*	*Behavioral health*	*Trauma-Informed System; Trauma-Focused CBT[d]*	*Y*	*Hybrid design case study*	*Quantitative*		*Study design, measurement, analysis, reporting*	*Single level inner context*	*Provider, patient*	*Y*	*Y*	*N*	*N*	*Y*	*Y*	*Adoption, Implementation, Penetration*	*1*	*1*	*2*	*0*	***4***
	Beidas RS, Edmunds J, Ditty M et al.[18]	*2014*	*Examine the association between adopter characteristics, individual perceptions of intra-organizational variables, and implementation outcomes for community therapists who received training and consultation in an EBP for youth anxiety.*	*USA*	*Community-based mental health clinics in one large US city*	*Mental health*	*Cognitive Behavioral Therapy (CBT)[e] for anxiety*	*N*	*Randomized implementation trial to test two therapist training conditions (training as usual versus computer training versus augmented training).*	*Quantitative*		*Study framing*	*Single level inner context*	*Provider*	*N*		*N*	*N*	*Y*	*Y*	*Fidelity, penetration*	*0*	*0*	*2*	*0*	***3***
	Beidas RS, Marcus S, Aarons GA et al.[19]	*2015*	*Examine the contribution of individual and organizational characteristics associated with therapist use of CBT, family therapy and psychodynamic therapy in a large-scale EBP implementation effort.*	*USA*	*19 agencies within large urban public mental health system in one large US city*	*Mental health*	*Cognitive Behavioral Therapy (CBT)[e] for youth psychiatric disorders; family therapy*	*N*	*cross-sectional*	*Quantitative*		*Study framing*	*Single level inner context*	*Provider*	*N*	*Y*	*N*	*N*	*Y*	*Y*	*Penetration*	*0*	*0*	*1*	*0*	***2***
	Skriner LC, Wolk CB, Stewart RE et al.[20]	*2018*	*Examine the impact of inner context factors, namely therapist and organization factors, on participation in Mental Health system policy mandate.*	*USA*	*22 behavioral health organizations within service system in one large US city*	*Mental health*	*Various evidence based mental health practices (CBT[e], PE,[f] DBTg[f], TF-CBT[d]) for adults and youth*	*N*	*Survey*	*Quantitative*		*Study framing, study design, measurement, analysis, reporting*	*Multilevel inner context*	*Organization, provider*	*N*	*Y*	*N*	*N*	*N*	*N*	*Y*	*0*	*1*	*2*	*0*	***3***

Project	Authors	Year	Objective	Country	Setting	Sector	EBP, Innovation or Intervention	Health focus reported (y/n)	Study design	Study methodology	Larger Study Design	Type of EPIS use	Level of data collection	Level of analysis	Outer context (y/n)	Inner context (y/n)	Innovation factors (y/n)	Bridging factors (y/n)	Imp Strategy (y/n)	Imp Strategy Reported (y/n)	Implement. Outcomes	Exploration	Preparation	Implementati	Sustainment	Depth (1: conceptual to 5: operational)
8			Examine the sustainment of six EBPs following fiscally mandated policy in the large public mental health services system of Los Angeles county.	USA ----	100 youth mental health agencies within one large US county	Mental Health	6 EBPs [Child-Parent Psychotherapy (CPP)[h]; Cognitive Behavioral Intervention for Trauma in School (CBITS)[i]; Managing and Adapting Practice (MAP)[j]; Seeking Safety[k]; Trauma- Focused Cognitive Behavioral Therapy (TF-CBT)[d]; Triple P Positive Parenting Program[l]]	N	Prospective observational	Mixed methods		Study framing, study design	Multilevel inner context	Organization, provider	Y	Y	Y	N	N	NA	Reach/ penetration, fidelity, maintenance	0	0	0	2	**3**
	Brookman-Frazee L, Stadnick N, Roesch S et al.[21]	*2016*	*Measuring Sustainment of Multiple Practices Fiscally Mandated in Children's Mental Health Services*	*USA*	*Youth mental health system in one large US county*	*Mental health*	6 EBPs [Child-Parent Psychotherapy (CPP)[h]; Cognitive Behavioral Intervention for Trauma in School (CBITS)[i]; Managing and Adapting Practice (MAP)[j]; Seeking Safety[k]; Trauma- Focused Cognitive Behavioral Therapy (TF-CBT)[d]; Triple P Positive Parenting Program[l]]	*N*	*Prospective*	*Quantitative*	*Larger Implementation effort to examine the sustainment of EBIs following fiscally mandated policy regarding EBIs*	*Study framing*	*Multilevel inner context*	*Organization, provider*	*N*	*Y*	*Y*	*N*	*N/A*	*N*	*Reach/ penetration*	*0*	*0*	*0*	*2*	***2***
	Lau AS, Brookman-Frazee L.[22]	*2016*	*To examine the sustainment of practices within a policy-driven, fiscally mandated, multiple EBP implementation effort in the large public mental health services system of Los Angeles county.*	*USA*	*100 agencies within mental health system in large US county*	*Mental health*	6 EBPs [Child-Parent Psychotherapy (CPP)[h]; Cognitive Behavioral Intervention for Trauma in School (CBITS)[i]; Managing and Adapting Practice (MAP)[j]; Seeking Safety[k]; Trauma- Focused Cognitive Behavioral Therapy (TF-CBT)[d]; Triple P Positive Parenting Program[l]]	*N*	*Observational - surveys and interviews*	*Mixed-Method*		*Study framing, study design*	*Multilevel inner context*	*Organization, provider*	*Y*	*Y*	*N*	*N*	*N*	*NA*	*Reach/ penetration, fidelity, maintenance*	*0*	*0*	*0*	*2*	***4***
9	Chamberlain P.[23]	2017	Describe the interventions and their implementation and preliminary results of two case studies.	USA	Two child welfare systems (one urban city private system to 5/35 agencies to 2000 children/year, other 4/12 rural regions in 24/95 counties: public system)	Child welfare	Keeping foster and kin parents supported and trained (KEEP)[m] and Parenting Through Change for Reunification (PTC-R)[n] and casework practice model (R3)[o]	N	Two case studies	Descriptive	Other papers on outcomes Chamberlain et al. (2016) and by Wulczyn and Feldman (in press)	Study framing, reporting	Multilevel inner and outer context	System, supervisors, providers	Y	Y	N	N	Y	Y	Fidelity, reach	0	2	2	0	**2**

Project	Authors	Year	Objective	Country	Setting	Sector	EBP, Innovation or Intervention	Health focus reported (y/n)	Study design	Study methodology	Larger Study Design	Type of EPIS use	Level of data collection	Level of analysis	Outer context (y/n)	Inner context (y/n)	Innovation factors (y/n)	Bridging factors (y/n)	Imp Strategy (y/n)	Imp Strategy Reported (y/n)	Implement. Outcomes	Exploration	Preparation	Implementati	Sustainment	Depth (1: conceptual to 5: operational)
10	Collins-Camargo C, Garstka TA.[24]	2014	Study whether implementation/use of evidence-informed practices was a function of organizational supervision and team goals that focuses on using data and outcome to inform workers on how well they are doing during the implementation of performance-based contracting and quality assurance systems within the context of a public/private partnership when out-of-home case management is provided by private contractors in three states in the US.	USA<br>----	Private agencies providing out-of-home care case management and residential treatment services	Child welfare	Evidence-informed practices for child welfare	N	Retrospective secondary data analysis	Quantitative	Y (see Collins-Camargo et al., 2007; Garstka, Collins-Carmargo, Hall, Neal, & Ensign, 2012)	Study design, measurement, analysis	Single level inner context	Organizational	N	Y	N	N	N	N	Use of evidence-informed practices (EIP)	0	0	2	0	**3**
11	Deane FP, Andresen R, Crowe TP *et al.*[25]	2014	Compare the effects of two different coaching approaches on the implementation and sustainment of a model of recovery-oriented service provision.	Australia	Four community-managed mental health organizations/clinics, representing 13 sites across four states of Australia	Mental Health	Collaborative Recovery Model[p] with ongoing coaching	N	prospective quasi-experimental	Quantitative		Study framing	Single level inner context	Provider	N	Y	N	N	Y	Y	Y	0	0	2	2	**2**
12	de Melo MNT, de Sá RMPF and Filho DAM.[26]	2016	Analyze the positive and negative factors related to the sustainability of the innovation to understand the processes related to the continuity of the innovative actions implemented.	Brazil	Schools in one Brazilian city	Public Health (Health promotion)	Brazilian School Food Program (PNAE)[q]	N	Case study	Qualitative		Study framing, analysis	Multilevel across outer and inner contexts	System and organization	Y	Y	N	Y	N	N	N	0	0	1	2	**1**
13	Drabble L, Lemon K, D'Andrade A *et al.*[27]	2013	Examine the process of establishing, implementing and sustaining a community-academic partnership between Child Welfare and University, including key factors impacting this process.	USA	School of Social Work and Child Welfare Agencies	Child welfare	Child Welfare Partnership for Research and Training[f]	N	Descriptive case study	Qualitative		Study design and reporting	Multilevel inner and outer context	System, organization	Y	Y	N	Y	Y	Y	N	2	2	2	2	**3**
14	Drahota A, Aarons GA, Stahmer AC.[28]	2012	Study protocol describing the development and pilot testing of a model of implementation to support the implementation of EBIs by Autism community providers.	USA	Community-based behavioral health agencies serving youth with autism spectrum disorder	Behavioral health	EBPs generally	N	Developmental and pilot test	Mixed method		Study design, data collection, measurement, reporting	Multilevel inner context	Organization, provider	N	Y	Y	Y	Y	Y	Feasibility, acceptability, fidelity	2	2	2	0	**2**
15	Edmunds JM, Read KL, Ringle VA *et al.*[29]	2014	Examine sustainment of clinicians providing an EBP two years following receiving training and consultation in the EBP.	USA	Mental health clinics	Mental Health	Cognitive Behavioral Therapy (CBT)[e]	N	Two year follow-up survey and interviews	Mixed method	Randomized trial to examine three training conditions	Study framing, measurement, reporting	Single level inner context	Provider	N	Y	N	N	Y	Y	Penetration, Sustainment (Fidelity)	0	0	0	2	**2**
16			Examine use of research evidence among leaders of county mental health, juvenile justice and child welfare systems participating in an RCT to scale up EBP implementation.	USA	Children's services in 51 counties across two US states	Juvenile justice, mental health, child welfare	Multidimenstional Foster Care (MTFC)[s]	N	Cross-sectional	Mixed methods	Stepped wedge RCT	Study framing, measurement, reporting	Multilevel outer and inner context		Y	Y	Y	Y	Y	N	Y	1	1	2	0	**4**
	Garcia AR, Kim M, Palinkas LA et al.[30]	*2016*	*Socio-contextual Determinants of Research Evidence Use in Public-Youth Systems of Care*	*USA*	*3 service settings in 37 counties across one US state*	*Child welfare, juvenile justice, and mental health*	*Multidimenstional Foster Care (MTFC)*[s]	*N*	*Retrospective cross-sectional*	*Quantitative*		*Study framing, reporting*	*Single level outer context*	*System*	*Y*	*Y*	*N*	*N*	*Y*	*N*	*Y*	*1*	*1*	*1*	*0*	**2**
	Palinkas L, Wu Q, Fuentes D et al.[31]	*2015*	*Examine use of research evidence among leaders of county mental health, juvenile justice and child welfare systems participating in an RCT to scale up EBP implementation for youth in foster care.*	*USA*	*Children's services in 51 counties across two US states*	*Juvenile justice, mental health, child welfare*		*N*	*Cross-sectional*	*Mixed method*		*Study framing, measurement*	*Multilevel outer and inner context*	*Organization*	*Y*	*Y*	*Y*	*Y*	*Y*	*N*	*Y*	*0*	*1*	*2*	*0*	**4**

Project	Authors	Year	Objective	Country	Setting	Sector	EBP, Innovation or Intervention	Health focus reported (y/n)	Study design	Study methodology	Larger Study Design	Type of EPIS use	Level of data collection	Level of analysis	Outer context (y/n)	Inner context (y/n)	Innovation factors (y/n)	Bridging factors (y/n)	Imp Strategy (y/n)	Imp Strategy Reported (y/n)	Implement. Outcomes	Exploration	Preparation	Implementati	Sustainment	Depth (1: conceptual to 5: operational)
17	Gadomski AM, Wissow LS, Palinkas L *et al.*[32]	2014	Provide insight into how to most effectively implement an implementation strategy for integrating MH and primary care, and how contextual factors influence sustainability.	USA	Primary care practices	Mental health	Primary Care Provider and Mental Health integration	N	cross-sectional using semi-structured interviews	Qualitative	Larger TEACH study	Study design, data collection, coding	Single level inner context	Provider	Y	Y	N	Y	Y	N	N	0	0	1	0	**2**
18	Gates LB, Hughes A, Kim DH.[33]	2015	Examine the impact of provider characteristics on the readiness to adopt a career development and employment intervention.	USA	Foster care through community NFPs and public child welfare agency	Child welfare	Career Development and Employment best practices	N	Retrospective survey	Quantitative		Study design, measurement	Multilevel inner context	Supervisor, provider	N	Y	N	N	N/A	N	N	2	2	0	0	**3**
19	Gleacher AA, Olin SS, Nadeem E *et al.*[34]	2016	Examine the factors and elucidate the interplay between barriers and facilitators that influenced the adoption and implementation of a technologically sophisticated measurement feedback system.	USA	Two community based mental health clinics	Mental health	Contextualized Feedback System (CFS)[t]	N	Semi-structured interviews	Mixed method	Quantitative uptake and impact study on client outcomes	Study framing, design, data collection, coding	Single level inner context	System, organization, provider, client	N	Y	Y	Y	Y	N	Adoption and implementation	0	2	2	0	**4**
20	Goldstein H, Olszewski A.[35]	2015	Describe the process of parallel intervention development with an implementation focus suing data based decision making of early literacy curriculum for preschoolers.	USA	Across early childhood education settings	Education	Supplemental early literacy curriculum	Y	Retrospective hybrid effectiveness/ implementation cluster RCT	Mixed method	Results paper, Greenwood et al. 2014	Reporting, interpretation	Multiple level outer and inner context	System, organizational, provider	Y	Y	Y	Y	Y	N	Feasbility, stages of implementation, fidelity	2	2	2	1	**3**
21	Green AE, Aarons GA.[36]	2011	Assess potential differences between administrators/ policymakers and those involved in direct practice regarding factors believed to be barriers or facilitating factors to EBP implementation.	USA	Community child and adolescent mental health services in one county in 1 US state	Mental health (child & adolescent)	EBP generally	N	Concept mapping, descriptive, cross-sectional	Mixed method		Reporting	Multilevel outer and inner context	All	Y	Y	Y	N	N/A	N	N	0	0	2	0	**3**
22	Guerrero EG, He A, Kim A *et al.*[37]	2014	Evaluate organizational factors associated with the implementation of contingency management treatment (CMT) and medication-assisted treatment (MAT) in substance abuse treatment (SAT) programs serving racial and ethnic minority communities.	USA	122 publicly-funded addiction health services programs in Public Health system in large US city	Substance Use (Public Health)	Contingency Management Treatment[u] and Medication-Assistant Treatment[v]	N	cross-sectional	Quantitative		Study framing, study design, data collection, analysis, reporting	Single level inner context	Organization, supervisor	Y	Y	N	N	N	N	Implementation	0	0	2	0	**3**
23	Guerrero EG, Andrews C, Harris L *et al.*[38]	2016	Evaluated the extent to which environmental and organizational characteristics influenced the likelihood of high coordination of mental health and public health services in addiction health services (AHS) in low-income racial and ethnic minority communities in Los Angeles, between 2011 and 2013.	USA	147 AHS outpatient programs serving low-income racial and ethnic minority communities in one large US city	Behavioral Health, Public Health, Primary Care	No intervention, rather coordinated mental health and public health services	N	Longitudinal study (two times points)	Mixed methods		Study framing, study design, measurement, analysis, reporting	Multilevel inner context	Organization, provider	Y	Y	Y	N	N	NA	N	0	0	1	0	**3**
24	Horwitz SM, Hurlburt MS, Goldhaber-Fiebert JD *et al.*[39]	2014	Examine use of existing resources as well as barriers and facilitators to exploration, adoption, and implementation of new EBPs in Child Welfare Agencies.	USA	Child welfare agencies	Child welfare	EBPs generally	N	Semi-structured interviews	Mixed method		Study design, data collection, interpretation	Single level inner context	Organization	N	N	N	N	N/A	N	Use	2	2	2	1	**2**
25	Humphrey A, Eastwood L, Atkins H, *et al.*[40]	2016	To describe commissioning and service structures enabling implementation of evidence-based cost-effective care as illustrated by the "1419" young people's service treating mild to moderate severity mental health difficulties in teenagers old 14 to 19 years.	UK	Mental Health	Mental health (child and adolescent)	Collaborative commissioning[w]	N	Cross-sectional observational hybrid design case study	Mixed method		Reporting	Single level inner context	Organizational, patient	Y	Y	Y	N	Y	Y	N	2	2	2	2	**3**

Project	Authors	Year	Objective	Country	Setting	Sector	EBP, Innovation or Intervention	Health focus reported (y/n)	Study design	Study methodology	Larger Study Design	Type of EPIS use	Level of data collection	Level of analysis	Outer context (y/n)	Inner context (y/n)	Innovation factors (y/n)	Bridging factors (y/n)	Imp Strategy (y/n)	Imp Strategy Reported (y/n)	Implement. Outcomes	Exploration	Preparation	Implementati	Sustainment	Depth (1: conceptual to 5: operational)
26	Hunter SB, Han B, Slaughter ME *et al.*[41]	2015	Examine factors associated with sustainment of the adolescent community reinforcement approach, an EBP, to address adolescent substance use.	USA	Community-based treatment organizations	Behavioral health	Adolescent community reinforcement approach[x]	N	Prospective - Interviews and surveys analyzed using discrete-time survival analysis	Mixed method		Study design, data collection, measurement	Multiple level inner context	Organization	Y	Y	Y	N	N/A	N	Sustainment (use, fidelity)	0	0	2	2	**1**
27	Kothari BH, McBeath B, Lamson-Siu E *et al.*[42]	2014	Present the development, delivery, cost, and feasibility of a novel sibling-focused intervention program.	USA	Foster care homes	Child welfare	Supporting Siblings in Foster Care (SIBS-FC)[y]	N	Prospective	Mixed-method	Prospective trial	Study framing	Multilevel inner context	Provider, client	N	N	N	Y	N/A	Y	Cost, fidelity	0	1	2	0	**1**
28	Kotte A, Hill KA, Mah AC *et al.*[43]	2016	Examine the implementation facilitators and barriers of a state-wide roll-out of a measurement feedback system (MFS) in a youth public mental health system.	USA	State-wide child and adolescent mental health department	Mental health	Measurement feedback systems (MFS)[z] for Ohio scale	N	Formative case study using semi-structured interviews	Qualitative		Study framing, reporting	Single level inner context	Organization, provider	Y	Y	Y	Y	N/A	N	N	1	1	2	1	**4**
29	Knight DK, Belenko S, Wiley T *et al.*[44]	2016	Study protocol describing large-scale hybrid implementation trial examining effectiveness of implementation strategies at promoting EBP use in Juvenile Justice and Behavioral Health Agencies across stages of implementation.	USA	Juvenile Justice and Behavioral Health Agencies	Juvenile Justice; Behavioral Health	EBPs	N	Hybrid design head-to-head cluster randomized trial with a phased rollout	Mixed method		Study design, data collection, measurement, analysis	Multilevel outer and inner context	Organization, patient	Y	Y	Y	Y	Y	Y	Fidelity; acceptability; sustainability	2	2	2	2	**5**
30	Lambert D, Richards T, Merrill T.[45]	2016	Examine contextual factors key across stages of implementation to Child Welfare implementation initiatives in three statewide efforts.	USA	Child welfare agencies across 3 US states	Child welfare	EBPs generally	N	Case studies	Qualitative		Study framing, design, reporting	Multilevel inner context	System	Y	Y	N	N	N/A	N	N	0	0	0	0	**2**
31	Leavy B, Kwak L, Hagströmer M *et al.*[46]	2017	Study protocol to test effectiveness of the clinical intervention, conduct a process evaluation and collect barriers and facilitators of implementation.	Sweden	4-6 rehabilitation clinics ranging from primary care to outpatient clinics specializing in neurological rehabilitation	Rehabilitation	HiBalance[aa]	Yes (study protocol)	Hybrid Type I, non-randomized control design	Mixed method	Protocol paper	Study design	Multiple level inner context	Provider, Patient	N	N	N	N	Y	Y	Fidelity, reach/penetration	2	2	2	0	**2**
32	Nadeem E, Weiss D, Olin SS *et al.*[47]	2016	Develop and pilot a brief, theory-based, manualized Learning Collaborative (LC) and determine whether community mental health clinics participating in the LC were more likely to implement a new practice being rolled-out in a statewide clinician training program compared to clinics participating in the state's training program as usual.	USA	12 child-serving clinics representing 10 community agencies in one large US city	Mental health (child and adolescent)	Managing and Adapting Practice (MAP)[j]	N	Randomized implementation study	Quantitative		Study framing, study design, reporting	Single level inner context	Organization, provider	N	Y	N	N	Y	Y	Fidelity, adoption, reach/ penetration	0	0	2	0	**2**
33	Nadeem E, Ringle VA.[48]	2016	Investigate the factors influencing the ongoing use of an EBP in an urban school district.	USA	1 urban school district	Education	Cognitive Behavioral Intervention for Trauma in Schools (CBITS)[i]	N	Retrospective using semi-structured interviews	Qualitative	Evaluation of CBITS implementat-ion	Reporting	Single level outer and inner context	System, organization	Y	Y	N	N	Y	Y	Y	0	0	2	2	**2**
34	Padwa H, Teruya C, Tran E *et al.*[49]	2016	Examine the implementation (inner and outer context factors) of integrated care protocols in three primary care organizations.	USA	Primary care clinics	Primary care	Project Care[bb]	N	Prospective and retrospective	Mixed method		Study design, data collection, measurement; analyses, reporting	Multilevel inner context	Organization	Y	Y	Y	Y	Y	Y	N	0	0	2	0	**4**

Project	Authors	Year	Objective	Country	Setting	Sector	EBP, Innovation or Intervention	Health focus reported (y/n)	Study design	Study methodology	Larger Study Design	Type of EPIS use	Level of data collection	Level of analysis	Outer context (y/n)	Inner context (y/n)	Innovation factors (y/n)	Bridging factors (y/n)	Imp Strategy (y/n)	Imp Strategy Reported (y/n)	Implement. Outcomes	Exploration	Preparation	Implementati	Sustainment	Depth (1: conceptual to 5: operational)
35	Palinkas LA, Fuentes D, Finno M *et al.*[50]	2014	To examine the role of inter-organizational collaboration in implementing new evidence- based practices for addressing problem behaviors in at-risk youth.	USA ----	Probation, mental health, and child welfare departments in 12 counties in one US state	Child Welfare	Multidimensional Treatment Foster Care (MTFC)[s]	N	Cross-sectional study	Qualitative	Stepped wedge, randomized controlled trial	Reporting	Single level outer context	System, organization	Y	Y	N	Y	Y	Y	N	0	0	2	0	**3**
36			Examine the clinical effectiveness, implementation process and perceptions of requirements for sustainment of a train-the-trainer implementation model of a behavioral intervention to reduce sexually transmitted infections among female sex workers in Mexico.	Mexico	Community-based reproductive health clinics across 12 Mexican cities	Public health	Mujer Segura (Healthy Woman)[cc]	Y	Hybrid Type 2	Mixed methods		Analysis, coding, reporting	Multilevel inner context	Organization, intervention	Y	Y	Y	N	Y	Y	Fidelity	0	0	2	2	**2**
	Patterson TL, Semple SJ, Chavarin CV et al.[51]	*2012*	*Implementation of an efficacious intervention for high risk women in Mexico: protocol for a multi-site randomized trial with a parallel study of organizational factors*	*Mexico*	*Community-based organizations across 12 Mexican cities*	*Public health*	*Mujer Segura (Healthy Woman)*[cc]	*Y*	*Hybrid, Type II*	*Mixed method*		*Analysis and coding*	*Multilevel inner context*	*Organization, intervention*	*N*	*Y*	*N*	*N*	*Y*	*Y*	*Fidelity*	*0*	*0*	*2*	*0*	**2**
	Palinkas LA, Chavarin CV, Rafful CM et al.[52]	*2015*	*Examine service provider perceptions of requirements for successful sustainment of an efficacious intervention for preventing HIV/AIDS and STIs in female sex workers.*	*Mexico*	*Community-based reproductive health clinics*	*Public health*	*Mujer Segura (Healthy Woman)*[cc]	*N*	*Follow-up semi-structured interviews*	*Qualitative*		*Reporting*	*Multilevel inner context*	*System, organization, provider, client, intervention*	*Y*	*Y*	*Y*	*N*	*Y*	*Y*	*N*	*0*	*0*	*2*	*2*	**2**
37	Peltzer K, Prado G, Horigian V *et al.*[53]	2016	Explore organizational and provider factors associated with prevention of mother-to-child HIV transmission implementation in South Africa.	South Africa	Community Health Centers	Public health	Prevention of mother-to-child HIV transmission (PMTCT)[dd]	N	Cross-sectional survey	Quantitative	Effectiveness clinical trial	Study framing	Multilevel inner context	Organizational, provider	N	Y	N	N	N/A	N	Acceptability, reach/penetration, fidelity	0	0	2	0	**1**
38			Examine factors impacting implementation of EBPs by substance use treatment providers serving American Indians/Alaska Natives (AI/ANs).	USA	Substance use disorder treatment programs serving American Indian/Alaskan Native communities	Substance use	EBPs generally	N	Survey and case-study	Mixed method		Study design; data collection, measurement, analysis reporting	Multilevel inner context	Organization, provider	N	Y	N	N	N/A	N	N	2	1	2	0	**2**
	Rieckmann T, Moore LA, Croy CD et al.[54]	*2016*	*A national study of American Indian and Alaska Native substance abuse treatment: provider and program characteristics*	*USA*	*Substance use disorder treatment centers*	*Substance use*	*EBPs generally*	*N*	*Survey*	*Quantitative*		*Study design, measurement, analysis, reporting*	*Multilevel inner context*	*Organization, provider*	*N*	*Y*	*N*	*N*	*Y*	*N*	*N*	*0*	*0*	*1*	*0*	**2**
	Moore LA, Aarons GA, Davis JH et al.[55]	*2015*	*Assess knowledge and attitudes towards EBPs among clinicians and clinical administrators working in treatment programs servicing American Indian/Alaskan Native communities.*	*USA*	*Substance use disorder treatment programs servicing American Indian/Alaskan Native communities*	*Substance use*	*EBPs generally*	*N*	*Observational - interviews and focus groups*	*Qualitative*		*Study design; data collection*	*Multilevel inner context*	*Provider*	*N*	*Y*	*N*	*N*	*N/A*	*N*	*N*	*2*	*1*	*0*	*0*	**2**
	Dickerson D, Moore LA, Rieckmann T et al.[56]	*2018*	*Examine contextual factors impacting implementation of MI by substance use treatment providers serving American Indians/Alaska Natives (AI/ANs).*	*USA*	*Substance use disorder treatment centers*	*Substance use*	*Motivational interviewing (MI)*[b]	*N*	*Retrospective survey*	*Quantitative*		*Study design, data measurement, analysis*	*Multilevel inner context*	*Organization, provider*	*N*	*Y*	*N*	*N*	*N/A*	*N*	*Y*	*0*	*0*	*2*	*0*	**2**

Project	Authors	Year	Objective	Country	Setting	Sector	EBP, Innovation or Intervention	Health focus reported (y/n)	Study design	Study methodology	Larger Study Design	Type of EPIS use	Level of data collection	Level of analysis	Outer context (y/n)	Inner context (y/n)	Innovation factors (y/n)	Bridging factors (y/n)	Imp Strategy (y/n)	Imp Strategy Reported (y/n)	Implement. Outcomes	Exploration	Preparation	Implementati	Sustainment	Depth (1: conceptual to 5: operational)
39	Rye M, Torres EM, Friborg O *et al.*[57]	2017	Describe the adaptation of a shortened version of the EBPAS and examine the psychometric properties of the shortened version in two samples.	USA & Norway	65 US mental health programs, Norwegian Psychological Association and Norwegian Nurses Organization	Mental health, psychology, nursing	EBP generally	N	cross-sectional measurement study	Quantitative		Study framing	Single level inner context	Provider	N	Y	N	N	N/A	N	EBP attitudes	0	0	0	0	**1**
40	Stroobants T Vanderfaeilli J, Andries C *et al.*[58]	2016	Examine the association between EBP knowledge, attitudes towards EBP, adoption of EBP and provider background characteristics.	Belgium	23 Home-visiting care organization in one Belgian city	Public health (child welfare)	EBP generally and empirically supported treatments (ESTs)	N	cross-sectional, descriptive, survey	Quantitative		Study framing, interpretation	Single level inner context	Provider	N	Y	N	N	N/A	N	Y	0	1	0	0	**1**
41	Walker SC, Whitener R, Trupin EW *et al.*[59]	2015	To explore how EBPs were currently perceived by tribes in Washington State and what kinds of policies and strategies tribal communities would recommend, if any, for the potential expansion of EBP.	USA	29 federally recognized and 7 non-federally recognized AI/AN tribes in one US State	Behavioral Health	Five EBPs	N	Cross-sectional study	Qualitative	Used findings from this study to inform next phase of planning an intervention to support implementation of EBPs in this community	Reporting	Multilevel inner context	System, community, individual, program	N	N	N	N	N	N	N	2	2	2	2	**2**
42	Wang B, Stanton B, Deveaux L *et al.*[60]	2017	This study examines factors influencing teachers' patterns of use of the intervention in the implementation phase and inner context attitudes and self-efficacy/comfort with the intervention. Implementation, the impact of teachers' initial implementation of FOYC, and subsequent delivery of the booster sessions on students' outcomes.	The Bahamas	114 government primary/ junior high schools in The Bahamas	Public Health	FOYC[ee]	Y	Prospective, longitudinal pre- and post-design survey	Quantitative		Study framing, study design, Measurement	Multilevel inner context	School, Teacher, student	N	Y	N	N	N	NA	Fidelity	0	0	2	0	**3**
43	Willging CE, Sommerfeld DH, Aarons GA *et al.*[61]	2014	Examine the impact of a statewide behavioral health reform on safety-net institutions, including impact on provider attitudes and reactions.	USA	14 Safety net institutions in a Behavioral Healthcare system in one US state	Behavioral health	ValueOptions New Mexico[ff]	N	Prospective Case Study	Mixed method	Prospective mixed-method study of behavioral health reform in New Mexico	Study design; sampling; data collection; reporting	Multilevel inner context	Organization	Y	Y	N	N		public–private partnership with a for-profit managed care company	N	0	0	2	0	**4**
44	Willging CE, Lamphere L, Rylko-Bauer B.[62]	2015	Evaluate and describe three initiatives In New Mexico (comprehensive community support services (CCSS), clinical homes (CHs), and core service agencies (CSAs)) as part of state-wide efforts to support people with serious mental illness.	USA	Behavioral health agencies	Mental health	Wraparound[gg]	N	descriptive study, using document review and qualitative interviews	Qualitative		Study framing, study design, data collection, coding	Multilevel inner and outer context	System, organizational	Y	Y	N	N	N	N	N	2	2	2	2	**4**
45	Willging CE, Green AE, Ramos MM.[63]	2016	Protocol of study aiming to test the 4-phases of implementation of a school nursing program to reduce LGBTQ Adolescent Suicide, including key cross-level implementation factors (student, provider, school, community) and youth outcomes.	USA	40 Publicly funded high schools in one US state	School-based Nursing	RLAS[hh]: Implementing School Nursing Strategies to Reduce LGBTQ Adolescent Suicide	Y (will be reported)	cluster randomized controlled trial, hybrid type I design	Mixed method		Study framing, study design, data collection, measurement, analysis, coding	Multilevel outer and inner context	Organization, provider	Y	Y	N	N	Y	Y	Fidelity	2	2	2	2	**5**

Project	Authors	Year	Objective	Country	Setting	Sector	EBP, Innovation or Intervention	Health focus reported (y/n)	Study design	Study methodology	Larger Study Design	Type of EPIS use	Level of data collection	Level of analysis	Outer context (y/n)	Inner context (y/n)	Innovation factors (y/n)	Bridging factors (y/n)	Imp Strategy (y/n)	Imp Strategy Reported (y/n)	Implement. Outcomes	Exploration	Preparation	Implementati	Sustainment	Depth (1: conceptual to 5: operational)
46	Williams JR, Blais MP, Banks D *et al.*[64]	2014	Examine the individual and organizational factors associated with adoption of motivational interviewing in community health organizations.	USA	92 Community Health Organizations/ Centers across the US	Public health	Motivational interviewing (MI)[b]	N	cross-sectional	Quantitative	RCT comparing 2 dissemination strategies among behavioral health and primary care providers.	Study framing, design, measurement	Multilevel inner context	Organization, supervisor provider	N	Y	N	N	N/A	N	Adoption	1	0	0	0	**2**
47	Stirman SW, Matza A, Gamarra J *et al.*[65]	2015	Examine contextual factors key to supporting sustainment of an EBP (Cognitive Therapy) in large, urban mental health system.	USA	Mental Health Clinics	Mental health	Cognitive Therapy[ii]	Y	Cross-sectional	Qualitative	Prospective observational	Study design, data collection, coding	Multilevel inner context	System, organizational	Y	N	Y	N	N	N	Fidelity	0	0	1	2	**3**
48	Wiltsey Stirman S, Finley EP, Shields N *et al.*[66]	2017	Protocol of study aiming to compare two implementation strategies (fidelity-oriented learning collaborative versus continuous quality improvement-oriented learning collaborative) promoting the sustainment of an EBP for posttraumatic stress disorder in community mental health clinics.	USA & Canada	Mental health clinics	Mental health	Cognitive Processing Therapy[jj]	Y (will be reported)	Type-III randomized hybrid design	Mixed method	Type-III mixed-methods randomized hybrid design	Data collection; coding	Multiple level inner context	Organization, Provider, Patient	N	Y	N	N	Y	Y	Fidelity; penetration; adaptation; costs	0	0	2	2	**2**
49	Zimmerman L, Lounsbury DW, Rosen CS *et al.*[67]	2016	Pilot test the Participatory Systems Dynamics Modeling (PSD) process, simulating implementation plans to improve EBP reach.	USA	1 Veterans Health Administration outpatient mental health system	Mental health	EBPs generally	N	Case study	Mixed method		Study framing	Single level inner context	System, organization, provider	Y	Y	Y	Y	Y	Y	Adoption, reach	0	0	2	0	**2**

Note: EBP, Innovation or Intervention

a. Safe Care: a behavioral and psychosocial, manualized and highly structured EBP that uses classic behavioral intervention techniques delivered through home-based training and education of caregivers developed to prevent child neglect and to reduce child maltreatment
b. Motivational interviewing (MI): a counseling approach to increase awareness of the potential problems, risks, and consequences of a behavior (e.g., substance use).
c. DECIDE: a patient activation and self-management intervention to teach ethnic/racial minority patients to take a more active role in their mental health care.
d. Trauma Focused Cognitive Behavioral Therapy (TF-CBT): manualized EBP to address trauma in youth.
e. Cognitive Behavioral Therapy (CBT): a mental health intervention that can be used to treat a range of psychiatric disorders.
f. Prolonged Exposure (PE): a mental health intervention that addresses trauma in adults.
g. Dialectical Behavior Therapy (DBT): a mental health intervention that targets emotional and behavioral dysregulation.
h. Child-Parent Psychotherapy (CPP): targets improving the caregiver-child relationship in the context of trauma experienced by the child.
i. Cognitive Behavioral Intervention for Trauma in School (CBITS): a mental health intervention delivered in a group format in schools to target trauma.
j. Managing and Adapting Practice (MAP): a suite of decision support tools to identify, select, and monitor EBP.
k. Seeking Safety: integrates CBT principles along with a present-focused, problem-oriented approach to address trauma and substance use.
l. Triple P Positive Parenting Program: behavioral parent training program delivered in a group or individual format.
m. Keeping foster and kin parents supported and trained (KEEP): a parenting model designed to teach parenting strategies that optimize child development, particularly within the child welfare system.
n. Parenting Through Change for Reunification (PTC-R): a parenting model adapted from the Parent Management Training Oregon for biological parents with children in foster care.
o. Casework practice model (R^3): a case-work practice model that integrates principles from KEEP and PTC-R into the daily interactions between casework supervisors and caseworkers.
p. Collaborative Recovery Model: a recovery-oriented model of service delivery.
q. Brazilian School Food Program (PNAE): National School Feeding Program in Brazil, an integrated food and nutrition security policy
r. Child Welfare Partnership for Research and Training: a community-engaged framework that includes a continuum of strategies aimed at community engagement in the research process.
s. Multidimensional Foster Care (MTFC):an EBP that targets out-of-home placements, youth arrests, violence, pregnancy, substance use and mental health problems.
t. Contextualized Feedback System (CFS): a measurement-feedback system to track clinical outcomes with the goal of improving service delivery
u. Contingency Management Treatment: a psychosocial intervention based on the principles of behavior modification to reduce substance use.
v. Medication-Assistant Treatment: a pharmacological intervention to target substance use and may be a supplement to a psychosocial intervention.
w. Collaborative commissioning: A collaboration between Cambridgeshire and Peterborough Clinical Commissioning Group, local authorities, and other partners which enables broad acceleration of service transformations
x. Adolescent community reinforcement approach: a behaviorally-based EBP to address adolescent substance use.
y. Supporting Siblings in Foster Care (SIBS-FC): a psychosocial sibling intervention program for pre-adolescent and adolescent youth in foster care.
z. Measurement Feedback Systems: a quality improvement tool to electronically track outcomes of health care services.
aa. HiBalance: a program that targets balance impairments in Parkinson's Disease to improve balance control and gait.
bb. Project Care: a pilot initiative that offered training, funding, and technical assistance to support integrated behavioral health care in primary care settings
cc. Mujer Segura (Healthy Woman): Psychoeducational intervention to promote self sex practices
dd. Prevention of mother-to-child transmission (PMTCT): a treatment protocol to prevent mother-to-child transmission of HIV.
ee. FOYC: An evidence-based, life skills curriculum designed to reduce risk taking behaviors related to HIV/STI transmission and teen pregnancy.
ff. ValueOptions New Mexico: Reform of publicly funded behavioral healthcare to reduce costs
gg. Wraparound: a treatment process that results in a unique set of community services and supports to facilitate individuals with mental illness to remain in their natural environments rather than institutions. This study defined Wraparound using: 3 initiatives: comprehensive community support services (CCSS), clinical homes (CHs), and core service agencies (CSAs)
hh. RLAS (Implementing School Nursing Strategies to Reduce LGBTQ Adolescent Suicide): an intervention model that builds on EPIS and the Dynamic Adaptation Process to implement EBP strategies in US high schools.
ii. Cognitive therapy: a mental health intervention that targets a range of psychiatric disorders.
jj. Cognitive processing therapy: a mental health intervention to target trauma in adults.

References

1. Aarons GA, Fettes DL, Sommerfeld DH, Palinkas LA: **Mixed methods for implementation research: Application to evidence-based practice implementation and staff turnover in community-based organizations providing child welfare services.** *Child Maltreatment* 2012, **17:**67-79.
2. Aarons GA, Sommerfeld DH: **Leadership, innovation climate, and attitudes toward evidence-based practice during a statewide implementation.** *J Am Acad Child Psy* 2012, **51:**423-431.
3. Green AE, Cafri G, Aarons G: **Job perceptions following statewide evidence-based treatment implementation.** *Journal of Children's Services* 2016, **11:**345-355.
4. Aarons GA, Green AE, Palinkas LA, Self-Brown S, Whitaker DJ, Lutzker JR, Silovsky JF, Hecht DB, Chaffin MJ: **Dynamic adaptation process to implement an evidence-based child maltreatment intervention.** *Implement Sci* 2012, **7:**1-9.
5. Aarons GA, Green AE, Willging CE, Ehrhart MG, Roesch SC, Hecht DB, Chaffin MJ: **Mixed-method study of a conceptual model of evidence-based intervention sustainment across multiple public-sector service settings.** *Implement Sci* 2014, **9:**183.
6. Willging CE, Green AE, Gunderson L, Chaffin M, Aarons GA: **From a "perfect storm" to "smooth sailing": Policymaker perspectives on implementation and sustainment of an evidence-based practice in two states.** *Child Maltreatment* 2015, **20:**24-36.
7. Aarons GA, Green AE, Trott E, Willging C, Torres EM, Ehrhart M, Roesch SC: **The roles of system and organizational leadership in system-wide evidence-based intervention sustainment: A mixed-method study.** *Adm Policy Ment Hlth* 2016, **43:**991-1008.
8. Willging C, Aarons GA, Trott E, Green AE, Finn CV, Ehrhart KH, Hecht D: **Contracting and procurement for evidence-based interventions in public-sector human services: A case study.** *Adm Policy Ment Hlth* 2016, **43:**675-692.
9. Aarons GA, Fettes DL, Hurlburt MS, Palinkas LA, Gunderson L, Willging CE, Chaffin MJ: **Collaboration, negotiation, and coalescence for interagency-collaborative teams to scale-up evidence-based practice.** *J Clin Child Adolesc* 2014, **43:**915-928.
10. Chaffin M, Hecht D, Aarons G, Fettes D, Hurlburt M, Ledesma K: **EBT fidelity trajectories across training cohorts using the Interagency Collaborative Team strategy.** *Adm Policy Ment Hlth* 2016, **43:**144-156.
11. Hurlburt M, Aarons GA, Fettes DL, Willging C, Gunderson L, Chaffin MJ: **Interagency Collaborative Team model for capacity building to scale-up evidence-based practice.** *Children and Youth Services Review* 2014, **39** 160-168.
12. Willging CE, Trott EM, Fettes D, Gunderson L, Green AE, Hurlburt MS, Aarons GA: **Research-supported intervention and discretion among frontline workers implementing home visitation services.** *Research on Social Work Practice* 2017, **27:**664-675.
13. Aarons GA, Ehrhart MG, Moullin JC, Torres EM, Green AE: **Testing the Leadership and Organizational Change for Implementation (LOCI) Intervention in substance abuse treatment: A cluster randomized trial study protocol.** *Implementation Science* 2017, **12:**29.
14. Ault-Brutus A, Lee C, Singer S, Allen M, Alegría M: **Examining implementation of a patient activation and self-management intervention within the context of an effectiveness trial.** *Adm Policy Ment Hlth* 2014, **41:**777-787.
15. Beidas RS, Aarons GA, Barg F, Evans A, Hadley T, Hoagwood K, Marcus S, Schoenwald S, Walsh L, Mandell DS: **Policy to implementation: evidence-based practice in community mental health—study protocol.** *Implement Sci* 2013, **9:**38.
16. Beidas RS, Stewart RE, Adams DR, Fernandez T, Lustbader S, Powell BJ, Aarons GA, Hoagwood KR, Evans AC, Hurford MO, et al: **A multi-level examination of stakeholder perspectives of implementation of evidence-based practices in a large urban publicly-funded mental health system.** *Adm Policy Ment Hlth* 2016, **43:**893-908.
17. Beidas RS, Adams DR, Kratz HE, Jackson K, Berkowitz S, Zinny A, Cliggitt LP, DeWitt KL, Skriner L, Evans A: **Lessons learned while building a trauma-informed public behavioral health system in the city of Philadelphia.** *Eval Program Plann* 2016, **59:**21-32.
18. Beidas RS, Edmunds J, Ditty M, Watkins J, Walsh L, Marcus S, Kendall P: **Are inner context factors related to implementation outcomes in cognitive-behavioral therapy for youth anxiety?** *Adm Policy Ment Hlth* 2014, **41:**788-799.
19. Beidas RS, Marcus S, Aarons GA, Hoagwood KE, Schoenwald S, Evans AC, Hurford MO, Hadley T, Barg FK, Walsh LM, et al: **Predictors of community therapists' use of therapy techniques in a large public mental health system.** *JAMA Pediatrics* 2015, **169:**374-382.
20. Skriner LC, Wolk CB, Stewart RE, Adams DR, Rubin RM, Evans AC, Beidas RS: **Therapist and organizational factors associated with participation in evidence-based practice initiatives in a large urban publicly-funded mental health system.** *The Journal of Behavioral Health Services & Research* 2018, **45:**174-186.
21. Brookman-Frazee L, Stadnick N, Roesch S, Regan J, Barnett M, Bando L, Innes-Gomberg D, Lau A: **Measuring sustainment of multiple practices fiscally mandated in children's mental health services.** *Adm Policy Ment Health* 2016, **43:**1009-1022.
22. Lau AS, Brookman-Frazee L: **The 4KEEPS study: identifying predictors of sustainment of multiple practices fiscally mandated in children's mental health services.** *Implement Sci* 2016, **11:**31.
23. Chamberlain P: **Toward creating synergy among policy, procedures, and implementation of evidence-based models in child welfare systems: two case examples.** *Clin Child Fam Psychol Rev* 2017, **20:**78-86.
24. Collins-Camargo C, Garstka TA: **Promoting outcome achievement in child welfare: predictors of evidence-informed practice.** *J Evid Based Soc Work* 2014, **11:**423-436.
25. Deane FP, Andresen R, Crowe TP, Oades LG, Ciarrochi J, Williams V: **A comparison of two coaching approaches to enhance implementation of a recovery-oriented service model.** *Adm Policy Ment Hlth* 2014, **41:**660-667.
26. de Melo MNT, de Sá RMPF, Filho DAM: **Sustainability of an innovative school food program: a case study in the northeast of Brazil.** *SciELO Public Health* 2016, **21:**1899-1908.
27. Drabble L, Lemon K, D'Andrade A, Donoviel B, Le J: **Child welfare partnership for research and training: a title IV-E university/community collaborative research model.** *Journal of Public Child Welfare* 2013, **7:**411-429.
28. Drahota A, Aarons GA, Stahmer AC: **Developing the Autism Model of Implementation for Autism spectrum disorder community providers: study protocol.** *Implement Sci* 2012, **7:**1-10.
29. Edmunds JM, Read KL, Ringle VA, Brodman DM, Kendall PC, Beidas RS: **Sustaining clinician penetration, attitudes and knowledge in cognitive-behavioral therapy for youth anxiety.** *Implement Sci* 2014, **9:**89.
30. Garcia AR, Kim M, Palinkas LA, Snowden L, Landsverk J: **Socio-contextual determinants of research evidence use in public-youth systems of care.** *Adm Policy Ment Hlth* 2016, **43:**569-578.
31. Palinkas LA, Qiaobing W, Fuentes D, Finno-Velasquez M, Holloway IW, Garcia A, Chamberlain P: **Innovation and the use of research evidence in youth-serving systems: a mixed-methods study.** *Child Welfare* 2015, **94:**57-85.
32. Gadomski AM, Wissow LS, Palinkas L, Hoagwood KE, Daly JM, Kaye DL: **Encouraging and sustaining integration of child mental health into primary care: interviews with primary care providers participating in Project TEACH (CAPES and CAP PC) in NY.** *Gen Hosp Psychiatry* 2014, **36:**555-562.
33. Gates LB, Hughes A, Kim DH: **Influence of staff attitudes and capacity on the readiness to adopt a career development and employment approach to services in child welfare systems.** *Journal of Public Child Welfare* 2015, **9:**323-340.
34. Gleacher AA, Olin SS, Nadeem E, Pollock M, Ringle V, Bickman L, Hoagwood K: **Implementing a measurement feedback system in community mental health clinics: a case study of multilevel barriers and facilitators.** *Adm Policy Ment Hlth* 2016:1-15.
35. Goldstein H, Olszewski A: **Developing a phonological awareness curriculum: reflections on an implementation science framework.** *J Speech Lang Hear Res* 2015, **58:**S1837-1850.
36. Green AE, Aarons GA: **A comparison of policy and direct practice stakeholder perceptions of factors affecting evidence-based practice implementation using concept mapping.** *Implement Sci* 2011, **6:**104.
37. Guerrero EG, He A, Kim A, Aarons GA: **Organizational implementation of evidence-based substance abuse treatment in racial and ethnic minority communities.** *Adm Policy Ment Hlth* 2014, **41:**737-749.
38. Guerrero EG, Andrews C, Harris L, Padwa H, Kong Y, M.S.W. KF: **Improving coordination of addiction health services organizations with mental health and public health services.** *J Subst Abuse Treat* 2016, **60:**45-53.
39. Horwitz SM, Hurlburt MS, Goldhaber-Fiebert JD, Palinkas LA, Rolls-Reutz J, Zhang J, Fisher E, Landsverk J: **Exploration and adoption of evidence-based practice by US child welfare agencies.** *Child Youth Serv Rev* 2014, **39:**147-152.
40. Humphrey A, Eastwood L, Atkins H, Vainre M, Lea-Cox C: **An exemplar of GP commissioning and child and adolescent mental health service partnership: Cambridge 1419 young people's service.** *Journal of Integrated Care* 2016, **24:**26-37.
41. Hunter SB, Han B, Slaughter ME, Godley SH, Garner BR: **Associations between implementation characteristics and evidence-based practice sustainment: a study of the Adolescent Community Reinforcement Approach.** *Implement Sci* 2015, **10:**173.
42. Kothari BH, McBeath B, Lamson-Siu E, Webb SJ, Sorenson P, Bowen H, Waid J, Bank L: **Development and feasibility of a sibling intervention for youth in foster care.** *Eval Program Plann* 2014, **47:**91-99.

43. Kotte A, Hill KA, Mah AC, Korathu-Larson PA, Au JR, Izmirian S, Keir SS, Nakamura BJ, Higa-McMillan CK: **Facilitators and barriers of implementing a measurement feedback system in public youth mental health.** *Adm Policy Ment Hlth* 2016, **43:**861-878.
44. Knight DK, Belenko S, Wiley T, Robertson AA, Arrigona N, Dennis M, Bartkowski JP, McReynolds LS, Becan JE, Knudsen HK, et al: **Juvenile Justice—Translational Research on Interventions for Adolescents in the Legal System (JJ-TRIALS): a cluster randomized trial targeting system-wide improvement in substance use services.** *Implementation Science* 2016, **11:**57.
45. Lambert D, Richards T, Merrill T: **Keys to implementation of child welfare systems change initiatives.** *Journal of Public Child Welfare* 2016, **10:**132-151.
46. Leavy B, Kwak L, Hagströmer M, Franzén E: **Evaluation and implementation of highly challenging balance training in clinical practice for people with Parkinson's disease: protocol for the HiBalance effectiveness-implementation trial.** *BMC Neurology* 2017, **17:**27.
47. Nadeem E, Weiss D, Olin SS, Hoagwood KE, Horwitz SM: **Using a theory-guided learning collaborative model to improve implementation of EBPs in a state children's mental health system: a pilot study.** *Adm Policy Ment Health* 2016, **43:**978-990.
48. Nadeem E, Ringle VA: **De-adoption of an evidence-based trauma intervention in schools: A retrospective report from an urban school district.** *School Mental Health* 2016, **8:**132-143.
49. Padwa H, Teruya C, Tran E, Lovinger K, Antonini VP, Overholt C, Urada D: **The implementation of integrated behavioral health protocols in primary care settings in Project Care.** *J Subst Abuse Treat* 2016, **62:**74-83.
50. Palinkas LA, Fuentes D, Finno M, Garcia AR, Holloway IW, Chamberlain P: **Inter-organizational collaboration in the implementation of evidence-based practices among public agencies serving abused and neglected youth.** *Adm Policy Ment Health* 2014, **41:**74-85.
51. Patterson TL, Semple SJ, Chavarin CV, Mendoza DV, Santos LE, Chaffin M, Palinkas L, Strathdee SA, Aarons GA: **Implementation of an efficacious intervention for high risk women in Mexico: protocol for a multi-site randomized trial with a parallel study of organizational factors.** *Implement Sci* 2012, **7:**105.
52. Palinkas LA, Chavarin CV, Rafful CM, Um MY, Mendoza DV, Staines H, Aarons GA, Patterson TL: **Sustainability of evidence-based practices for HIV prevention among female sex workers in Mexico.** *PLOS ONE* 2015, **10:**e0141508.
53. Peltzer K, Prado G, Horigian V, Weiss S, Cook R, Sifunda S, Jones D: **Prevention of mother-to-child transmission (PMTCT) implementation in rural community health centres in Mpumalanga province, South Africa.** *Journal of Psychology in Africa* 2016, **26:**415-418.
54. Rieckmann T, Moore LA, Croy CD, Novins DK, Aarons G: **A national study of American Indian and Alaska Native substance abuse treatment: provider and program characteristics.** *J Subst Abuse Treat* 2016.
55. Moore LA, Aarons GA, Davis JH, Novins DK: **How do providers serving American Indians and Alaska Natives with substance abuse problems define evidence-based treatment?** *Psychol Serv* 2015, **12:**92-100.
56. Dickerson D, Moore LA, Rieckmann T, Croy CD, Venner K, Moghaddam J, Gueco R, Novins DK: **Correlates of motivational interviewing use among substance use treatment programs serving American Indians/Alaska Natives.** *The Journal of Behavioral Health Services & Research* 2018, **45:**31-45.
57. Rye M, Torres EM, Friborg O, Skre I, Aarons GA: **The Evidence-based Practice Attitude Scale-36 (EPBAS-36): a brief and pragmatic measure of attitudes to evidence-based practice validated in Norwegian and U.S. samples.** *Implement Sci* 2017.
58. Stroobants T, Vanderfaeillie J, Andries C, Van Holen F: **Youth care workers' perspectives on and adoption of evidence-based practice.** *Children and Youth Services Review* 2016, **71:**299-307.
59. Walker SC, Whitener R, Trupin EW, Migliarini N: **American Indian perspectives on evidence-based practice implementation: results from a statewide Tribal Mental Health Gathering.** *Adm Policy Ment Health* 2015, **42:**29-39.
60. Wang B, Stanton B, Deveaux L, Lunn S, Rolle G, Adderley R, Poitier M, Koci V, Marshall S, Gomez P: **Multi-year school-based implementation and student outcomes of an evidence-based risk reduction intervention.** *Implement Sci* 2017, **12:**16.
61. Willging CE, Sommerfeld DH, Aarons GA, Waitzkin H: **The effects of behavioral health reform on safety-net institutions: A mixed-method assessment in a rural state.** *Adm Policy Ment Hlth* 2014, **41:**276-291.
62. Willging CE, Lamphere L, Rylko-Bauer B: **The transformation of behavioral healthcare in New Mexico.** *Adm Policy Ment Hlth* 2015, **42:**343-355.
63. Willging CE, Green AE, Ramos MM: **Implementing school nursing strategies to reduce LGBTQ adolescent suicide: a randomized cluster trial study protocol.** *Implement Sci* 2016, **11:**145.
64. Williams JR, Blais MP, Banks D, Dusablon T, Williams WO, Hennessy KD: **Predictors of the decision to adopt motivational interviewing in community health settings.** *The Journal of Behavioral Health Services & Research* 2014, **41:**294-307.
65. Stirman SW, Matza A, Gamarra J, Toder K, Xhezo R, Evans AC, Hurford M, Beck AT, Crits-Christoph P, Creed T: **System-level influences on the sustainability of a cognitive therapy program in a community behavioral health network.** *Psychiatr Serv* 2015, **66:**734-742.
66. Wiltsey Stirman S, Finley EP, Shields N, Cook J, Haine-Schlagel R, Burgess JF, Dimeff L, Koerner K, Suvak M, Gutner CA, et al: **Improving and sustaining delivery of CPT for PTSD in mental health systems: a cluster randomized trial.** *Implement Sci* 2017, **12:**32.
67. Zimmerman L, Lounsbury DW, Rosen CS, Kimerling R, Trafton JA, Lindley SE: **Participatory system dynamics modeling: increasing stakeholder engagement and rrecision to improve implementation planning in systems.** *Adm Policy Ment Health* 2016, **43:**834-849.
